# Ethnobotanical insights into the traditional food plants of the Baiku Yao community: a study of cultural significance, utilization, and conservation

**DOI:** 10.1186/s13002-024-00691-y

**Published:** 2024-05-16

**Authors:** Binsheng Luo, Yuanming Tong, Yujing Liu, Ying Zhang, Yixin Qin, Renchuan Hu

**Affiliations:** 1 Lushan Botanical Garden, Jiangxi Province and Chinese Academy of Sciences, Lushan, 332900 China; 2 Guangxi Key Laboratory of Traditional Chinese Medicine Quality Standards, Guangxi Institute of Chinese Medicine and Pharmaceutical Science, Nanning, 530022 China; 3 College of Life Sciences, Nanjing Agriculture University, Nanjing, 210000 China

**Keywords:** Ethnobotany, Baiku Yao, Edible plants, Cultural significance, Traditional knowledge

## Abstract

**Background:**

The Baiku Yao, primarily residing in Guangxi and Guizhou provinces of China, is a distinctive branch of the Yao ethnic group, known for their profound cultural preservation and unique ethnobotanical knowledge. This study investigates the Baiku Yao community’s utilization of traditional food plants, focusing on the relationship between their dietary practices and the local biodiversity within their mountainous living environment. It aims to illuminate the cultural significance and survival strategies embedded in their ethnobotanical knowledge, highlighting the potential for sustainable living and biodiversity conservation.

**Methods:**

Through ethnobotanical surveys, key informant interviews, and quantitative analysis techniques such as the cultural food significance index (CFSI) and relative frequency of citations (RFC), this research systematically documents the diversity and cultural importance of edible plants in the Baiku Yao community. The study assesses how these plants contribute to the community’s diet, traditional medicine, and overall cultural practices.

**Results:**

A total of 195 traditional edible plants were documented, belonging to 142 genera and 68 families, with a significant concentration in certain families such as Asteraceae, Rosaceae, and Fabaceae. The Baiku Yao diet prominently features herbaceous plants, with wild (103 species) and cultivated (89 species) varieties as diverse food sources. They utilize various plant parts, particularly fruits and leaves, for multiple purposes, including nutrition, medicine, and fodder. Their processing techniques, from raw to fermented, showcase a rich culinary tradition and emphasize a holistic use of plants for enhancing diet and health in a concise overview. The RFC and CFSI analyses reveal a deep cultural reliance on a variety of plant species, with a notable emphasis on vegetables, fruits, spices, and medicinal herbs. Specific plants like *Zingiber officinale*, *Zea mays*, and *Oryza sativa* were highlighted for their high cultural significance. The study also uncovers the multifunctional use of these plants, not only as food but also for medicinal purposes, fodder, and other cultural applications, reflecting the Baiku Yao’s profound ecological wisdom and their harmonious coexistence with nature.

**Conclusion:**

The findings emphasize the rich ethnobotanical knowledge possessed by the Baiku Yao, underscoring the importance of documenting, safeguarding, and transmitting this invaluable traditional knowledge. This study contributes to a deeper understanding of cultural heritage and biodiversity conservation, advocating for concerted efforts to protect such traditional practices against the threats of modernization and cultural erosion.

## Introduction

Edible plants serve not only as a primary food source for many local ethnic groups and communities but also represent a significant area of research in ethnobotany [1, 2]. In specific circumstances, such as natural disasters or food shortages, certain wild plants are considered ‘famine foods’ and play a crucial role in sustaining food security [3]. The study and conservation of these food plants, as well as the associated traditional knowledge, are vital for understanding and utilizing biodiversity.

According to statistics, there are over 50,000 edible plant species globally. However, data from the United Nations Food and Agriculture Organization (FAO) indicate that just 15 major crops satisfy 90% of the world’s energy needs. This fact reveals humanity’s deep reliance on a limited number of crops and highlights the significant value and unutilized potential of nature as a resource repository [4]. Nevertheless, the rapid development of urbanization and modern agriculture is leading to the gradual decline of traditional farming methods, affecting the utilization rate of wild edible plants and resulting in changes in related traditional cultural knowledge and ethno-ecosystems [5, 6].

During the COVID-19 pandemic, many remote areas globally, particularly mountainous communities, faced a series of challenges of food supply shortages and rising prices [7]. This situation increased local communities’ reliance on and consumption of wild edible plants, proving the critical role of traditional edible plants in maintaining food security [8]. The Baiku Yao of China, a group dependent on traditional farming and wild collecting, demonstrated unique resilience and adaptability in addressing food challenges during the pandemic.

The Baiku Yao, primarily located in the Baxu Yao Township and Lihu Yao Township in Nandan County, Guangxi Province, and the Yaoshan Yao Township in Libo County, Guizhou Province, is a distinct branch of the Yao ethnic group in China, with a total population about 40,000 [9]. They got this name because of the men’s traditional knee-length white trousers. Baiku Yao was recognized by UNESCO as one of the ethnic groups with the most intact cultural preservation, and they are also referred to as a ‘living fossil of human civilization’ [9]. The Baiku Yao primarily live deep in the mountains, often in limestone depressions, hillsides, or mountaintops. They have preserved their traditional farming culture, which has continued for thousands of years, mainly growing crops such as corn, soybeans, rice, cotton, and oilseeds [9]. Their dietary habits and food sources reflect the rich diversity of their culture.

Recent studies have highlighted the Baiku Yao’s extensive traditional knowledge of plant utilization. For instance, Hu et al. [10] revealed their use of plants and original techniques in traditional clothing dyeing. Luo and others have focused on the application of traditional fodder plants and veterinary plants among the Baiku Yao communities [11, 12]. Recent research has also begun to focus on their edible plants. In the latest studies, Luo et al. studied the nutritional value and economic potential of a local food plant, *Lindera pulcherrima* var. *attenuata*, providing rich data and theoretical support for understanding their food culture and sustainable development [13]. However, the ethnobotany of Baiku Yao’s food plant culture remains an important but insufficiently explored field. Additionally, the Baiku Yao, a community that transitioned directly from a primitive to a modern society, has long inhabited the edges of the mountainous and limited land areas of the Yunnan–Guizhou Plateau. We are very curious about how they have adapted to the challenging local conditions and how they manage their limited food supplies.

Thus, this study aims to resolve the above question and fill this research gap by systematically investigating the practice of applying edible plants in Baiku Yao communities, revealing this crucial ‘puzzle piece.’ This research surveyed the diversity of traditional edible plants used by the Baiku Yao, analyzed the characteristics of their dietary culture, and explored their survival strategies in the mountainous regions on the edge of the Yunnan–Guizhou Plateau and their interaction with the environment. Additionally, this study will assess the functions and values of these traditional edible plants, exploring those with potential for development. The results can provide positive insights and academic references for future studies on these unutilized food plants.

## Methods

### Study sites

**Fig. 1:**
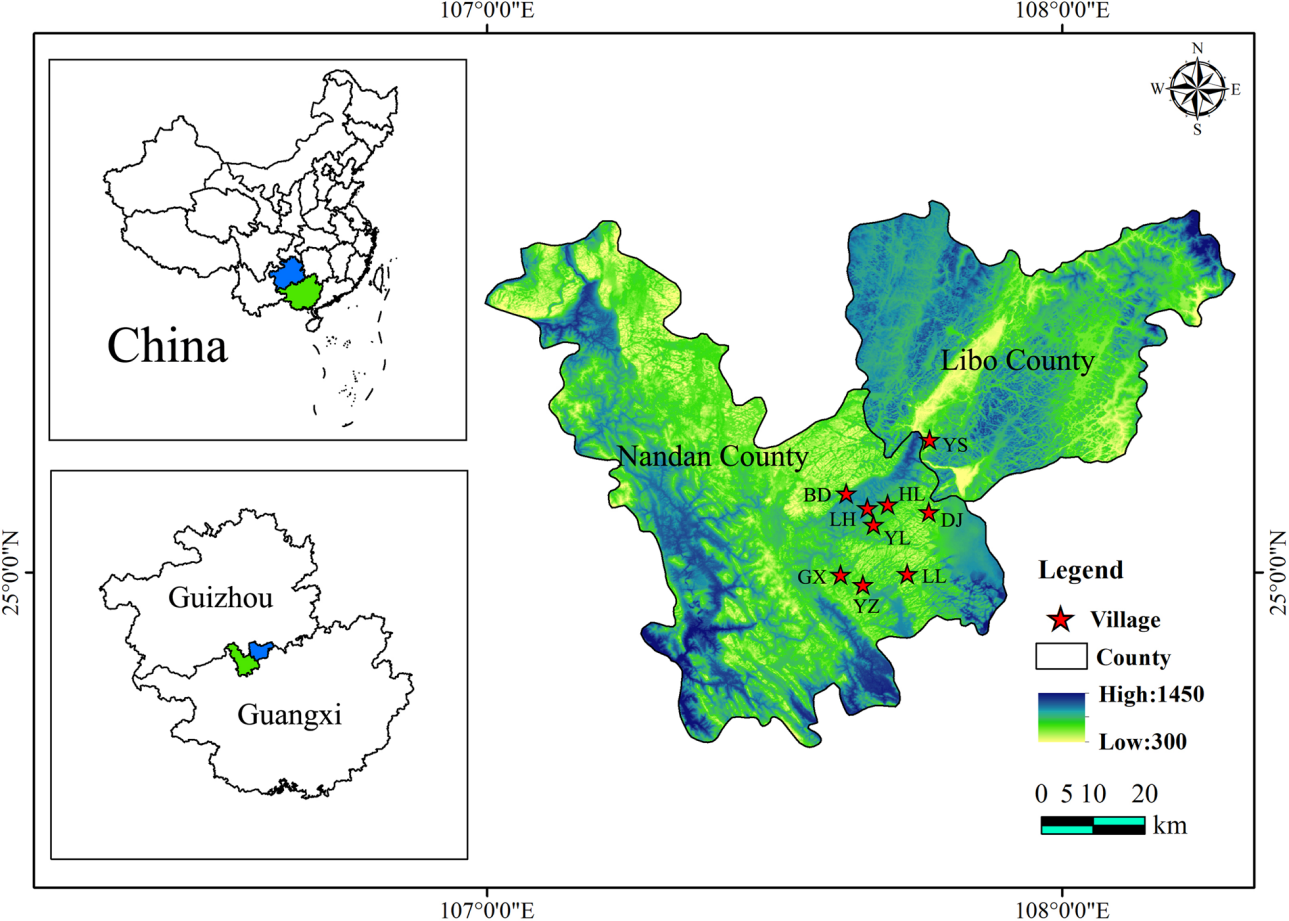
The study sites. (LH: Lihu Community, HL: Huaili Village, DJ: Dongjia Village, YL: Yaoli Village, and BD: Badi Village, YZ: Yaozhai Village, LL: Lile Village, GX: Guanxi Village, YS: Yaoshan Village)

Based on a review of Baiku Yao literature, in conjunction with preliminary survey results and recommendations from the Baiku Yao Ethnic Museum staff, we selected the Lihu Yao Township (Lihu Community, Huaili Village, Dongjia Village, Yaoli Village, and Badi Village), Baxu Yao Township (Yaozhai Village, Lile Village, and Guanxi Village) in Nandan County in Guangxi Province, and Yaoshan Yao Village in Yaoshan Township, Libo County in Guizhou Province in China, as our research sites (see Fig. 1, Table 1). These selected locations are primary settlements of the Baiku Yao, where traditional culture is exceptionally well-preserved, facilitating our data collection efforts. 90% of the population locally are Baiku Yao people who speak their own ethnic language. From 2020 to 2023, we conducted eight ethnobotanical surveys across different villages of the Baiku Yao ethnic group, covering all four seasons.

**Table 1:** The situation of study sites

No.	Village name	Longitude	Latitude	Altitude (m)	Ecological environment	Population
1	Huaili Village	107° 41′ 58.72″	25° 06′ 59.05″	756	Limestone karst depressions, mainly lithosolic soil, predominantly subtropical evergreen broadleaf forest	3169
2	Lihu Community	107° 39′ 39.96″	25° 06′ 42.56″	572	Limestone karst depressions, mainly lithosolic soil, predominantly secondary *Pinus massoniana* forest	1699
3	Dongjia Village	107° 46′ 25.88″	25° 06′ 06.10″	822	Limestone karst depressions, mainly lithosolic soil, predominantly subtropical evergreen broadleaf forest	3997
4	Yaoli Village	107° 40′ 19.87″	25° 04′ 57.51″	759	Limestone karst depressions, mainly lithosolic soil, predominantly secondary *Pinus massoniana* forest	5904
5	Badi Village	107° 37′ 29.04″	25° 08′ 14.43″	742	Limestone karst depressions, mainly lithosolic soil, predominantly subtropical evergreen broadleaf forest	3340
6	Yaozhai Village	107° 39′ 10.62″	24° 58′ 39.29″	805	Limestone karst depressions, mainly lithosolic soil, predominantly secondary *Pinus massoniana* forest	1492
7	Lile Village	107° 43′ 50.56″	24° 59′ 49.52″	699	Limestone karst depressions, mainly lithosolic soil, predominantly subtropical evergreen broadleaf forest	2824
8	Guanxi Village	107° 35′ 24.75″	24° 59′ 55.63″	802	Limestone karst depressions, mainly lithosolic soil, predominantly secondary *Pinus massoniana* forest	2809
9	Yaoshan Village	107° 46′ 07.56″	25° 13′ 46.70″	516	Limestone karst depressions, mainly lithosolic soil, predominantly secondary *Pinus massoniana* forest	3177

The relevant data in the table are sourced from interviews with local government officials, with the statistical year ending in December 2023

### Ethnobotanical interviews

The current study mainly utilized ethnobotanical methodologies, including key informant interviews supplemented by semi-structured and informal interviews. We meticulously recorded the ethnographic information (age, gender, occupation, etc.) of participants and information about the investigated species, including local Yao names, parts used, applications, resource types, processing methods, and precautions [14]. In our investigations within Baiku Yao communities, we selected 186 interviewees in total. Fifty-five key informants were recommended by local government officials, staff of the Baiku Yao Ethnic Museum, and village leaders, all possessing extensive knowledge and experience in plant utilization. An additional 131 participants were selected using the random selection method and snowball sampling at local markets or within Baiku Yao villages, with ages ranging from 20 to 82 years. Among the 186 interviewees, there were 84 males and 102 females; the selection of interviewees exhibited a degree of randomness. Thus, the gender ratio is not representative. We also employed participatory observations and field investigations to record how local people process food plants and collect voucher specimens or photograph them from the wild [14].

The identification and analysis of these voucher specimens and their photographs were conducted using resources such as Flora of China, Guangxi Plant Records, and relevant online databases (http://www.iplant.cn, http://www.cfh.ac.cn/, https://www.gbif.org/, http://www.nsii.org.cn/2017/home.php, https://www.worldfloraonline.org/). This process facilitated the documentation of food plants in Baiku Yao communities and laid the groundwork for research and analysis of food plant resources. The voucher specimens are stored at the Herbarium of the Guangxi Zhuang Autonomous Region Institute of Traditional Chinese Medicine (GXMI).

### Data analysis

The cultural food significance index (CFSI) encompasses a broad range of criteria to assess the cultural importance of specific wild edible plants. The CFSI is determined using a formula provided by Pieroni [15], designed to evaluate the cultural value of wild edibles:

$${\text{CFSI}} = {\text{QI}} \times {\text{AI}} \times {\text{FUI}} \times {\text{PUI}} \times {\text{MFFI}} \times {\text{TSAI}} \times {\text{FMRI}} \times 10^{ - 2}$$

The CFSI includes quotation frequency (QI, frequency of quotation index: the number of people who mentioned a plant among all informants), availability (AI, availability index: divided into very common (4.0), common (3.0), average (2.0) and uncommon (1.0), Correction Index: Widespread (=), In some places (− 0.5), In a particular place (− 0.1)), typology of the used parts (PUI, parts used index: divided into Whole plant (2.0), Leaf (2.0), Root (2.0), Branches and leaves (2.0), Tender branches and leaves (1.5), Fruit (1.50), Rhizome (1.5), Young shoot (1.25), Seed (1.0), Tender stem (1.0), Bark (1.0), Tender leaf (0.75), Flower (0.75), frequency of use (FUI, frequency of utilization index: divided into more than once a week (5.0), once a week (4.0), once a month (3.0), more than once a year but less than once a month (2.0), once a year (1.0) and unused for nearly 30 years (0.5)), kind and a number of food uses (MFFI, multifunctional food use index: divided into raw food and cold salad (1.5), boiling, stewing and seasoning (1.0), special purpose and condiments (0.75) and raw food as snacks (0.50), taste appreciation (TSAI, taste score appreciation index: divided into excellent (10.0), very good (9.0), good (7.5), fair (6.5), poor (5.5) and very poor (4.0)) and perceived role as food medicine (FMRI, food-medicinal role index: divided into very high (as medicinal food: 5.0), high (as medicine to treat a certain disease: 4.0), moderately high (very healthy food: 3.0), moderately low (healthy food, unknown efficacy: 2.0) and unknown or possibly toxic (1.0)).

As inferred from the formula, the value of the cultural food significance index (CFSI) is determined by a combination of factors: the frequency of mentions of a specific plant, its commonness, frequency of use, parts utilized, multifunctionality, taste evaluation, and its role in food-medicine practices. A higher CFSI value indicates a more significant role of the plant in dietary practices, thereby enabling preliminary identification of edible plants that are highly accepted and valued by people [15]. Applying the CFSI enables us to recognize the cultural significance of wild edible plants, highlighting their role in local diets and their potential for broader nutritional and medicinal uses [15, 16].

This research additionally employed the relative citation frequency (RFC) as a method for statistical examination. The RFC values were determined through the formula provided in [17]:

$${\text{RFC}} = \frac{{{\text{FCs}}}}{N}$$

Here, FCs denote the aggregate count of citations for each plant species by all respondents, while N is the total count of respondents [17]. The range of RFC values is from 0 to 1, where larger values suggest a more vital linkage between the food plant and the everyday existence of the Baiku Yao [18].

## Results and discussion

### Diversity analysis of Baiku Yao traditional edible plants

In this survey, a total of 195 traditional edible plants of the Baiku Yao were documented, belonging to 142 genera and 68 families (Table 2). The taxonomic distribution of Baiku Yao’s edible plants revealed a concentration in certain families and a dispersion among genera (Table 3). The distribution among families shows that the diversity of these edible plants is concentrated in relatively few families. Specifically, there are 14 families with multiple species (≥ 5 species), comprising 114 species, accounting for 58.46% of all the edible plant species identified. Additionally, 18 families with a few species (2–4 species) comprise 44 species, making up 23.08% of the total. The remaining 36 families each contain only one edible plant species, summing up to 36 species, which is 18.46% of the total. In terms of genera, the distribution is more dispersed. There are 3 genera with multiple species (≥ 5 species), containing 17 species in total, representing 8.72% of all identified edible plant species. The genera with a few species (2–4 species) include 28 genera, containing 67 species, which is 34.36% of the total. The most common are monotypic genera, with 111, each having only one species, adding up to 111 species in total and accounting for 56.92% of the total.

**Table 2:** List of edible plants used by Baiku Yao

Scientific names	Local name	Family name	Life form	Resource types	Collection season	Used parts	Process methods	Edible used	Other uses	QI	AI	FUI	PUI	MFFI	TSAI	FMRI	CFSI	Inventory No
*Ipomoea batatas* (Linnaeus) Lamarck	Yan du	Convolvulaceae	Liana	Cultivated	Whole year	Leaf, tuberous root	Boil, stir-fry	Vegetable, staple food	Medicine, fodder	158	4.0	4.0	3.00	2.50	10.0	3.0	5688.00	HRC705
*Zanthoxylum armatum* Candolle	Bi rua	Rutaceae	Shrub	Wild	Fall	Fruit, tender sprout	Seasoning, boil	Spice, vegetable	Medicine, veterinary drug	125	3.0	3.5	2.25	2.00	10.0	5.0	2953.13	HRC114
*Glycine max* (L.) Merr.		Fabaceae	Herb	Cultivated	Summer, fall	Fruit	Boil, stir-fry	Vegetable	Fodder	176	3.5	4.0	1.50	2.50	10.0	3.0	2772.00	HRC341
*Morus alba* Linnaeus		Moraceae	Tree	Cultivated	Spring, fall	Tender sprout, fruit	Boil, stir-fry	Vegetable, fruit	Medicine, fodder	172	4.0	2.0	2.75	2.50	6.5	4.0	2459.60	HRC108
*Raphanus sativus* L.	Guo bo a	Brassicaceae	Herb	Cultivated	Fall, winter, spring	Tuberous root, leaf	Stir-fry, boil, eat directly	Vegetable, fruit	Medicine	160	3.0	2.0	3.00	2.50	7.5	4.0	2160.00	HRC1048
*Musa basjoo* Siebold & Zuccarini	Wu sao	Musaceae	Herb	Cultivated	Whole year	Stem, fruit	Wine making, eat directly	Wine making, fruit	Medicine, fodder	180	3.0	3.0	2.50	1.75	10.0	3.0	2126.25	HRC732
*Zingiber officinale* Roscoe	A qiang	Zingiberaceae	Herb	Cultivated	Whole year	Tuber	Seasoning	Spice		186	4.0	5.0	1.50	1.00	7.5	5.0	2092.50	HRC1051
*Cucumis sativus* L.		Cucurbitaceae	Herb	Cultivated	Summer, fall	Fruit	Stir-fry, salad, eat directly	Vegetable, fruit	Medicine	152	4.0	2.0	1.50	3.00	9.0	4.0	1969.92	HRC336
*Pisum sativum* L.	Da a lai	Fabaceae	Herb	Cultivated	Whole year	Fruit, tender sprout	Boil, stir-fry	Vegetable		173	3.0	2.0	2.25	2.50	10.0	3.0	1751.63	HRC1040
*Capsicum annuum* L.	E biu	Solanaceae	Shrub	Cultivated	Whole year	Fruit	Seasoning	Vegetable		183	4.0	5.0	1.50	1.00	10.0	3.0	1647.00	HRC1046
*Allium hookeri* Thwaites	Ya ma li	Liliaceae	Herb	Cultivated	Whole year	Leaf	Boil, stir-fry	Vegetable		138	3.0	3.5	1.50	2.50	10.0	3.0	1630.13	HRC1023
*Plantago asiatica* Linnaeus	Ya du ma	Plantaginaceae	Herb	Wild	Spring	Whole plant	Boil, stir-fry	Vegetable	Medicine, fodder	165	4.0	2.0	1.50	2.50	6.5	5.0	1608.75	HRC453
*Amaranthus tricolor* L.		Amaranthaceae	Herb	Cultivated	Spring	Tender sprout	Boil, stir-fry	Vegetable	Medicine, fodder	130	4.0	3.0	1.50	2.50	9.0	3.0	1579.50	HRC501
*Perilla frutescens* (Linnaeus) Britton	Wo bai mi	Lamiaceae	Herb	Cultivated	Whole year	Leaf	Seasoning	Spice	Medicine, veterinary drug	170	4.0	3.0	1.50	1.00	10.0	5.0	1530.00	HRC833
*Zea mays* L.		Poaceae	Herb	Cultivated	Fall	Seed	Boil, wine making	Staple food, wine making	Fodder	186	4.0	5.0	1.00	1.75	7.5	3.0	1464.75	HRC339
*Brassica rapa* var. oleifera de Candolle		Brassicaceae	Herb	Cultivated	Fall, winter, spring	Tender sprout	Boil, stir-fry	Vegetable		180	4.0	2.0	1.50	2.50	9.0	3.0	1458.00	HRC940
*Houttuynia cordata* Thunberg		Saururaceae	Herb	Wild	Whole year	Whole plant	Stir-fry, salad	Vegetable	Medicine, fodder	152	3.5	3.0	1.50	2.00	7.5	4.0	1436.40	HRC111
*Aster ageratoides* Turcz	Ya bu sai	Asteraceae	Herb	Wild	Spring	Tender sprout	Boil, stir-fry	Vegetable	Medicine, fodder	135	3.5	2.0	1.50	2.50	9.0	4.0	1275.75	HRC778
*Cannabis sativa* L.		Cannabaceae	Herb	Cultivated	Winter	Seed	Boil	Soup ingredient	Medicine, fodder	176	4.0	3.5	1.00	1.00	10.0	5.0	1232.00	HRC86
*Brassica juncea* (Linnaeus) Czernajew	Wo bie ou	Brassicaceae	Herb	Cultivated	Fall, winter, spring	Leaf	Boil, stir-fry	Vegetable		160	3.0	3.0	1.50	2.50	7.5	3.0	1215.00	HRC1175
*Allium fistulosum* L.	Wo mie zha	Liliaceae	Herb	Cultivated	Whole year	Leaf	Seasoning	Spice		136	4.0	3.5	1.50	1.00	10.0	4.0	1142.40	HRC1049
*Solanum americanum* Miller	Wo guo	Solanaceae	Herb	Wild	Spring	Tender sprout	Boil, stir-fry	Vegetable	Medicine, fodder	122	3.0	2.0	1.50	2.50	7.5	5.0	1029.38	HRC831
*Lactuca sativa* var. ramosa Hort.		Asteraceae	Herb	Cultivated	Fall, winter, spring	Leaf	Boil, stir-fry	Vegetable		142	3.5	2.0	1.50	2.50	9.0	3.0	1006.43	HRC1034
*Agastache rugosa* (Fisch. et Mey.) O. Ktze.		Labiatae	Herb	Cultivated	Whole year	Tender sprout	Seasoning	Spice		132	3.5	3.5	1.50	1.00	10.0	4.0	970.20	HRC989
*Solanum lycopersicum* L.		Solanaceae	Herb	Cultivated	Fall, winter	Fruit	Stir-fry	Vegetable	Medicine	170	4.0	3.0	1.50	1.00	10.0	3.0	918.00	HRC804
*Vigna umbellata* (Thunb.) Ohwi et Ohashi	Da mian	Fabaceae	Herb	Cultivated	Fall, winter	Seed	Boil, stir-fry	Vegetable		116	3.5	3.0	1.00	2.50	7.5	4.0	913.50	HRC1099
*Mentha canadensis* Linnaeus	Yang gei nie	Lamiaceae	Herb	Cultivated, wild	Whole year	Tender sprout	Seasoning	Spice	Medicine	168	3.0	3.0	1.50	1.00	10.0	4.0	907.20	HRC312
*Allium sativum* L.	Wo mie hou	Liliaceae	Herb	Cultivated	Whole year	Whole plant	Seasoning	Vegetable		142	4.0	3.5	1.50	1.00	10.0	3.0	894.60	HRC1052
*Colocasia esculenta* (L.) Schott.	Wo niang e	Araceae	Herb	Cultivated	Fall, winter	全株	Boil, stir-fry	Vegetable, staple food		115	3.0	3.0	1.50	2.50	7.5	3.0	873.28	HRC1176
*Luffa aegyptiaca* Miller		Cucurbitaceae	Liana	Cultivated	Summer	Fruit	Boil, stir-fry	Vegetable	Medicine	134	3.0	2.0	1.50	2.50	9.0	3.0	814.05	HRC128
*Cucurbita moschata* (Duch. ex Lam.) Duch. ex Poiret	Gao	Cucurbitaceae	Herb	Cultivated	Fall, winter	Fruit	Stir-fry	Vegetable	Fodder	160	4.0	3.0	1.50	1.00	9.0	3.0	777.60	HRC951
*Cymbopogon citratus* (D. C.) Stapf	Xia yi	Poaceae	Herb	Cultivated	Whole year	Leaf	Seasoning, boil	Spice, herbal tea	Medicine	92	2.0	3.0	1.50	2.00	9.0	4.0	596.16	HRC711
*Anredera cordifolia* (Tenore) Steenis		Basellaceae	Liana	Wild	Spring, fall	Tender sprout	Boil, stir-fry	Vegetable	Fodder	88	2.5	2.0	1.50	2.50	9.0	4.0	594.00	HRC841
*Acorus tatarinowii* Schott		Araceae	Herb	Cultivated	Whole year	Leaf	Seasoning	Spice		120	3.0	3.0	1.50	1.00	9.0	4.0	583.20	HRC1090
*Emilia sonchifolia* (L.) DC.		Asteraceae	Herb	Wild	Spring	Tender sprout	Boil, stir-fry	Vegetable	Medicine, fodder	81	2.5	2.0	1.50	2.50	7.5	5.0	569.53	HRC304
*Rosa laevigata* Michaux.	Ni kao	Rosaceae	Shrub	Wild	Fall	Seed coat	Eat directly, sugar making	Fruit	Medicine, veterinary drug	75	3.5	2.0	1.50	1.50	9.0	5.0	531.56	HRC11
*Oryza sativa* L.	Cuo	Poaceae	Herb	Cultivated	Fall	Seed	Boil	Staple food, wine making	Dye, fodder, religious celebration	186	2.5	5.0	1.00	1.00	7.5	3.0	523.13	HRC648
*Broussonetia papyrifera* (Linnaeus) L’Heritier ex Ventenat	Wo bi jie	Moraceae	Tree	Cultivated, wild	Spring, fall	Tender sprout, fruit	Boil, stir-fry, eat directly	Vegetable, fruit	Medicine, fodder	42	4.0	2.0	3.00	2.50	6.5	3.0	491.40	HRC875
*Amaranthus spinosus* L.	A niang	Amaranthaceae	Herb	Cultivated	Spring, summer	Tender sprout	Boil, stir-fry	Vegetable	Fodder	80	3.0	2.0	1.50	2.50	9.0	3.0	486.00	HRC947
*Pteridium aquilinum* var. latiusculum (Desv.)Underw.ex Heller		Dennstaedtiaceae	Herb	Wild	Spring, winter	Tender sprout, root	Stir-fry, roast	Vegetable		130	4.0	1.0	2.75	1.50	7.5	3.0	482.63	HRC57
*Artemisia indica* Willd.	Wa huo	Asteraceae	Herb	Wild	Spring	Tender sprout	Boil, stir-fry	Vegetable	Fodder	68	3.0	2.0	1.50	2.50	7.5	4.0	459.00	HRC877
*Solanum melongena* L.	Ya gu	Solanaceae	Herb	Cultivated	Whole year	Fruit	Stir-fry	Vegetable		180	3.0	2.0	1.50	1.00	9.0	3.0	437.40	HRC1045
*Dicliptera chinensis* (L.) Juss.		Acanthaceae	Herb	Wild	Spring, summer	Tender sprout	Boil, stir-fry	Vegetable	Medicine, fodder	73	3.0	2.0	1.50	2.50	6.5	4.0	427.05	HRC89
*Dioscorea japonica* Thunb.		Dioscoreaceae	Liana	Wild	Winter, spring	Tuber	Roast, boil, stir-fry	Staple food, vegetable		67	2.5	2.0	1.50	3.00	9.0	3.0	407.03	HRC856
*Persicaria odorata* (Lour.) Soják		Polygonaceae	Herb	Wild	Whole year	Tender sprout	Seasoning	Spice	Medicine	95	2.0	3.5	1.50	1.00	10.0	4.0	399.00	HRC280
*Eriobotrya japonica* (Thunb.) Lindl.	Bī bò lè	Rosaceae	Tree	Cultivated, wild	Spring, summer	Fruit	Eat directly	Fruit	Medicine	143	4.0	2.0	1.50	0.50	9.0	5.0	386.10	HRC204
*Allium ramosum* L.		Liliaceae	Herb	Wild	Whole year	Whole plant	Stir-fry	Vegetable		118	3.0	2.0	1.50	1.00	9.0	4.0	382.32	HRC985
*Saccharum officinarum* L.		Poaceae	Herb	Cultivated	Whole year	Stem	Eat directly	Fruit		160	2.5	3.0	1.50	0.50	10.0	4.0	360.00	HRC1042
*Crassocephalum crepidioides* (Benth.) S. Moore	Wo suan gao	Asteraceae	Herb	Wild	Spring	Tender sprout	Boil, stir-fry	Vegetable	Medicine, fodder	60	3.5	2.0	1.50	2.50	7.5	3.0	354.38	HRC824
*Dioscorea persimilis* Prain et Burkill		Dioscoreaceae	Herb	Wild	Winter, spring	Tuberous root	Stew, stir-fry	Vegetable, staple food		81	2.5	2.0	1.50	2.50	7.5	3.0	341.72	HRC342
*Camellia oleifera* Abel.		Theaceae	Tree	Cultivated	Fall	Seed	Press oil	Edible oil		92	2.0	4.0	1.00	1.00	9.0	5.0	331.20	HRC1476
*Leucocasia gigantea* (Blume) Schott	Wo bie	Araceae	Herb	Cultivated	Whole year	Petiole	Stir-fry	Vegetable	Fodder	115	3.5	2.0	1.00	1.50	9.0	3.0	326.03	HRC828
*Cryptotaenia japonica* Hassk.	Wo duo wu	Apiaceae	Herb	Wild	Spring	Tender sprout	Boil, stir-fry	Vegetable	Medicine, fodder	75	2.5	2.0	1.50	2.50	7.5	3.0	316.41	HRC850
*Gynura japonica* (Thunb.) Juel.		Asteraceae	Herb	Wild	Spring	Tender sprout	Boil, stir-fry	Vegetable	Medicine, veterinary drug, fodder	68	2.0	2.0	1.50	2.50	7.5	4.0	306.00	HRC800
*Allium chinense* G. Don		Liliaceae	Herb	Wild	Whole year	Whole plant	Seasoning	Spice		82	3.0	2.0	1.50	1.00	10.0	4.0	295.20	HRC107
*Bambusa chungii* McClure	A mo a	Poaceae	Herb	Cultivated	Summer	Bamboo shoot	Stir-fry	Vegetable	Religious celebration	142	3.0	2.0	1.00	1.50	7.5	3.0	287.55	HRC1032
*Brassica oleracea* var. capitata Linnaeus		Brassicaceae	Herb	Cultivated	Winter, spring	Leaf	Stir-fry	Vegetable		142	3.0	2.0	1.50	1.00	7.5	3.0	287.55	HRC1033
*Oenanthe javanica* (Bl.) DC.		Apiaceae	Herb	Wild	Spring	Tender sprout	Boil, stir-fry	Vegetable	Fodder	82	2.5	1.0	1.50	2.50	9.0	4.0	276.75	HRC973
*Amorphophallus konjac* K. Koch	Gei niao ge bie	Araceae	Herb	Cultivated	Fall, winter	Tuber	Stir-fry	Vegetable	Medicine	90	3.0	2.0	1.50	1.50	7.5	3.0	273.38	HRC943
*Bidens pilosa* L.	Wo zong	Asteraceae	Herb	Wild	Spring, summer	Tender sprout	Boil, stir-fry	Vegetable	Veterinary drug, fodder	35	4.0	2.0	1.50	2.50	6.5	4.0	273.00	HRC821
*Eleutherococcus trifoliatus* (Linnaeus) S. Y. Hu		Araliaceae	Shrub	Cultivated	Spring	Tender sprout	Boil, stir-fry	Vegetable	Medicine	56	2.5	2.0	1.50	2.50	6.5	4.0	273.00	HRC642
*Hovenia acerba* Lindl.	Bi zha	Rhamnaceae	Tree	Cultivated	Fall	Fruit	Eat directly, infuse with liquor	Fruit		97	2.5	2.0	1.50	1.25	7.5	4.0	272.81	HRC1169
*Centella asiatica* (L.) Urban		Apiaceae	Herb	Wild	Spring, summer, fall	Whole plant	Boil	Vegetable	Medicine	68	3.5	2.0	1.50	1.00	7.5	5.0	267.75	HRC1068
*Hibiscus syriacus* L.		Malvaceae	Shrub	Cultivated	Spring	Tender sprout	Boil	Vegetable	Fodder	110	3.0	2.0	1.50	1.00	9.0	3.0	267.30	HRC950
*Vigna unguiculata* (L.) Walp.	Da (dao)	Fabaceae	Liana	Cultivated	Summer, fall	Fruit	Stir-fry	Vegetable		132	3.0	2.0	1.50	1.00	7.5	3.0	267.30	HRC1025
*Alpinia japonica* (Thunb.) Miq.	A giang zuo	Zingiberaceae	Herb	Cultivated	Whole year	Leaf	Seasoning	Spice		80	3.0	2.0	1.50	1.00	9.0	4.0	259.20	HRC1139
*Cichorium intybus* L.		Asteraceae	Herb	Cultivated	Fall, winter	Leaf	Boil, stir-fry	Vegetable		60	2.5	2.0	1.50	2.50	7.5	3.0	253.13	HRC1054
*Dioscorea fordii* Prain et Burkill		Dioscoreaceae	Liana	Wild	Winter, spring	Tuberous root	Boil, stir-fry	Vegetable, staple food		62	2.0	2.0	1.50	2.50	9.0	3.0	251.10	HRC1479
*Prunus persica* L.		Rosaceae	Tree	Cultivated	Spring, fall	Fruit	Eat directly	Fruit	Medicine, religious celebration	186	3.0	2.0	1.50	0.50	10.0	3.0	251.10	HRC240
*Lactuca sativa* var. angustata Irish ex Bremer		Asteraceae	Herb	Cultivated	Winter, spring	Leaf	Boil, stir-fry	Vegetable		59	2.5	2.0	1.50	2.50	7.5	3.0	248.91	HRC1021
*Gynura bicolor* (Willd.) DC.		Asteraceae	Herb	Cultivated	Spring, summer, fall	Tender sprout	Boil, stir-fry	Vegetable		55	2.0	2.0	1.50	2.50	7.5	4.0	247.50	HRC939
*Basella alba* L.		Basellaceae	Herb	Cultivated	Spring, summer	Tender sprout	Boil, stir-fry	Vegetable		58	2.5	2.0	1.50	2.50	7.5	3.0	244.69	HRC1112
*Senna tora* (Linnaeus) Roxburgh		Fabaceae	Herb	Wild	Spring, fall	Tender sprout, fruit	Boil	Vegetable, staple food		55	3.0	2.0	2.75	1.00	6.5	4.0	235.95	HRC1478
*Piper sarmentosum* Roxb.		Piperaceae	Herb	Cultivated	Whole year	Leaf	Seasoning	Spice		86	2.0	2.0	1.50	1.00	9.0	5.0	232.20	HRC1114
*Lycium chinense* Miller		Solanaceae	Shrub	Cultivated	Spring, fall	Tender sprout, fruit	Boil	Vegetable, fruit	Medicine, fodder	69	1.5	2.0	3.00	1.00	9.0	4.0	223.56	HRC949
*Crepidiastrum denticulatum* (Houttuyn) Pak & Kawano		Asteraceae	Herb	Wild	Spring, summer	Tender sprout	Boil, stir-fry	Vegetable	Medicine, fodder	42	3.0	2.0	1.50	2.50	7.5	3.0	212.63	HRC909
*Acorus gramineus* Soland.		Araceae	Herb	Cultivated	Whole year	Leaf	Stew	Spice	Medicine, veterinary drug	65	2.0	3.0	1.50	1.00	9.0	4.0	210.60	HRC808
*Momordica subangulata* Bl.		Cucurbitaceae	Herb	Cultivated	Summer, fall, winter	Fruit	Boil, stir-fry	Vegetable		61	1.5	2.0	1.50	2.50	7.5	4.0	205.88	HRC1088
*Prunus salicina* Lindl.		Rosaceae	Tree	Cultivated	Spring, summer	Fruit	Eat directly	Fruit		186	3.0	2.0	1.50	0.50	7.5	3.0	188.33	HRC984
*Chenopodium album* L.		Amaranthaceae	Herb	Wild	Spring	Tender sprout	Boil, stir-fry	Vegetable	Medicine, fodder	40	2.0	2.0	1.50	2.50	7.5	4.0	180.00	HRC70
*Buddleja officinalis* Maxim.	Bo se	Buddlejaceae	Shrub	Wild	Spring	花	Steam	Food coloring	Medicine, dye	80	2.5	2.0	1.50	1.00	10.0	3.0	180.00	HRC79
*Senna occidentalis* (Linnaeus) Link		Fabaceae	Herb	Wild	Spring, fall	Tender sprout, fruit	Boil	Vegetable, staple food		43	2.5	2.0	2.75	1.00	7.5	4.0	177.38	HRC1477
*Psidium guajava* L.		Myrtaceae	Shrub	Wild	Fall	Fruit	Eat directly	Fruit		130	3.0	2.0	1.50	0.50	7.5	4.0	175.50	HRC1488
*Citrus maxima* (Burm.) Merr.		Rutaceae	Tree	Cultivated	Fall, winter	Fruit	Eat directly	Fruit	Religious celebration	85	3.0	2.0	1.50	0.50	9.0	5.0	172.13	HRC987
*Hylocereus undatus* (Haw.) Britt. et Rose		Cactaceae	Herb	Wild	Spring	花, fruit	Stew, eat directly	Vegetable, fruit	Fodder	62	1.5	2.0	2.25	1.50	9.0	3.0	169.49	HRC964
*Canna indica* ‘Edulis’		Cannaceae	Herb	Cultivated	Winter	Tuber	Boil	Staple food	Fodder	80	3.0	2.0	1.50	1.00	7.5	3.0	162.00	HRC106
*Dioscorea polystachya* Turczaninow		Dioscoreaceae	Liana	Cultivated	Winter, spring	Tuberous root	Boil, stir-fry	Vegetable, staple food	Medicine	48	2.0	2.0	1.50	2.50	7.5	3.0	162.00	HRC1480
*Phaseolus vulgaris* L.	Dao mu	Fabaceae	Liana	Cultivated	Whole year	Fruit	Stir-fry	Vegetable		115	2.0	2.0	1.50	1.00	7.5	3.0	155.25	HRC1024
*Juglans regia* L.		Juglandaceae	Tree	Cultivated	Fall	Fruit	Eat directly	Fruit	Medicine	130	3.5	2.0	1.50	0.50	7.5	3.0	153.56	HRC701
*Curcuma longa* L.	Gang	Zingiberaceae	Herb	Wild	Whole year	Tuberous root	Steam	Food coloring	Medicine, dye	70	2.5	2.0	1.50	0.75	7.5	5.0	147.66	HRC552
*Toona sinensis* (A. Jussieu) M. Roemer		Meliaceae	Tree	Cultivated	Spring	Tender sprout	Boil, stir-fry	Vegetable	Medicine, dye	36	4.0	1.0	1.50	2.50	9.0	3.0	145.80	HRC322
*Vitis vinifera* L.	Bi gai	Vitaceae	Liana	Cultivated	Fall	Fruit	Eat directly, wine making	Fruit		82	2.0	2.0	1.50	1.25	7.5	3.0	138.38	HRC1075
*Castanea mollissima* Blume	Bi yi	Fagaceae	Tree	Cultivated	Fall	Seed	Eat directly	Fruit	Medicine	110	4.0	2.0	1.00	0.50	10.0	3.0	132.00	HRC684
*Pyrus pyrifolia* (Burm. F.) Nakai		Rosaceae	Tree	Cultivated	Fall	Fruit	Eat directly	Fruit		125	3.0	2.0	1.50	0.50	7.5	3.0	126.56	HRC983
*Lablab purpureus* (L.) Sweet		Fabaceae	Herb	Cultivated	Summer, fall	Fruit	Stir-fry	Vegetable		61	2.5	2.0	1.50	1.00	9.0	3.0	123.53	HRC403
*Capsella bursa-pastoris* (L.) Medic.		Brassicaceae	Herb	Wild	Spring	Tender sprout	Boil, stir-fry	Vegetable	Medicine, fodder	24	2.5	2.0	1.50	2.50	9.0	3.0	121.50	HRC971
V*itis heyneana* Roem. et Schult	Bi gai nong	Vitaceae	Liana	Wild	Winter	Fruit	Wine making	Fruit, wine making		130	2.5	2.0	1.50	0.75	5.5	3.0	120.66	HRC776
*Clausena lansium* (Lour.) Skeels		Rutaceae	Tree	Cultivated	Fall	Fruit	Eat directly	Fruit		88	3.5	2.0	1.50	0.50	6.5	4.0	120.12	HRC1178
*Litsea cubeba* (Loureiro) Persoon		Lauraceae	Tree	Wild	Fall	Fruit	Seasoning	Spice	Medicine	53	2.5	2.0	1.50	1.00	7.5	4.0	119.25	HRC1486
*Myrica rubra* Siebold & Zuccarini	Di ma	Myricaceae	Tree	Wild	Spring	Fruit	Eat directly	Fruit	Medicine	186	3.0	1.0	1.50	0.50	9.0	3.0	113.00	HRC651
*Illicium verum* Hook. f.		Schisandraceae	Tree	Cultivated	Fall	Fruit	Seasoning	Spice		62	1.5	2.0	1.50	1.00	10.0	4.0	111.60	HRC1484
*Hypolepis punctata* (Thunb.) Mett.	Heng zhe	Dennstaedtiaceae	Herb	Wild	Spring	Tender sprout	Stir-fry	Vegetable		53	2.5	1.0	1.50	1.50	9.0	4.0	107.33	HRC991
*Rubus rosifolius* Smith		Rosaceae	Herb	Wild	Spring	Fruit	Eat directly	Fruit	Medicine	132	4.0	1.0	1.50	0.50	9.0	3.0	106.92	HRC679
*Pueraria montana* var. lobata (Willdenow) Maesen & S. M. Almeida ex Sanjappa & Predeep		Fabaceae	Liana	Wild	Fall, winter, spring	Tuberous root	Boil	Staple food		70	3.0	1.0	1.50	1.00	6.5	5.0	102.38	HRC1489
*Leonurus japonicus* Houttuyn	Zu ei gai min	Lamiaceae	Herb	Wild	Spring	Tender sprout	Boil	Vegetable	Medicine	37	3.0	2.0	1.50	1.00	7.5	4.0	99.90	HRC364
*Campanumoea javanica* Bl.	Ya wa	Campanulaceae	Herb	Wild	Fall, winter	Tuberous root, fruit	Stew	Tonic, fruit	Medicine	35	1.5	1.0	3.00	1.50	9.0	4.5	95.68	HRC685
*Dioscorea subcalva* Prain & Burkill		Dioscoreaceae	Liana	Wild	Fall, winter	Tuber	Roast	Staple food	Medicine, dye	80	3.0	2.0	1.50	1.00	6.5	2.0	93.60	HRC97
*Gynostemma pentaphyllum* (Thunb.) Makino		Cucurbitaceae	Liana	Wild	Spring	Tender sprout	Boil	Vegetable	Medicine, fodder	41	2.0	2.0	1.50	1.00	7.5	5.0	92.25	HRC1483
*Diospyros oleifera* Cheng	Pi bei	Ebenaceae	Tree	Cultivated	Fall, winter	Fruit	Eat directly	Fruit		105	2.5	2.0	1.50	0.50	7.5	3.0	88.59	HRC1013
*Talinum paniculatum* (Jacq.) Gaertn.		Talinaceae	Herb	Wild	Spring	Tender sprout	Boil, stir-fry	Vegetable	Fodder	26	3.0	1.0	1.50	2.50	7.5	4.0	87.75	HRC975
*Litsea pungens* Hemsl.	Zuo ke	Lauraceae	Shrub	Wild	Fall, winter	Fruit	Seasoning	Spice		40	2.0	2.0	1.50	1.00	9.0	4.0	86.40	HRC987
*Gardenia jasminoides* Ellis		Rubiaceae	Shrub	Wild	Fall, winter	Fruit	Steam	Food coloring	Dye	71	3.0	1.0	1.50	0.75	9.0	4.0	86.27	HRC250
*Imperata cylindrica* (Linnaeus) Raeuschel		Poaceae	Herb	Wild	Whole year	Stem	Eat directly	Fruit	Medicine, religious celebration	76	4.0	1.0	1.50	0.50	7.5	5.0	85.50	HRC480
*Physalis angulata* L.		Solanaceae	Herb	Wild	Spring	Tender sprout	Boil	Vegetable	Medicine	35	2.5	2.0	1.50	1.00	6.5	5.0	85.31	HRC1487
*Sechium edule* (Jacq.) Swartz		Cucurbitaceae	Liana	Cultivated	Fall, winter	Fruit	Stir-fry	Vegetable		62	2.0	2.0	1.50	1.00	7.5	3.0	83.70	HRC986
*Rhus chinensis* Mill.	Dang ji bu	Anacardiaceae	Tree	Wild	Fall, winter	Fruit	Soak in cold water	Lye water		81	3.5	1.0	1.50	0.75	6.5	4.0	82.92	HRC993
*Codonopsis pilosula* (Franch.) Nannf.		Campanulaceae	Liana	Wild	Whole year, fall	Tuberous root, fruit	Stew	Tonic, fruit		19	1.5	2.0	3.00	1.00	9.0	5.0	76.95	HRC760
*Sorghum bicolor* (L.) Moench	A yong	Poaceae	Herb	Cultivated	Fall	Fruit	Boil, wine making	Staple food, wine making	Fodder	60	2.5	1.0	1.50	1.75	6.5	3.0	76.78	HRC948
*Beta vulgaris* var. cicla L.	Ya she	Chenopodiaceae	Herb	Cultivated	Winter, spring	Leaf	Boil	Vegetable		43	3.0	2.0	1.50	1.00	6.5	3.0	75.47	HRC1061
*Liquidambar formosana* Hance	Yin mei	Hamamelidaceae	Tree	Wild	Spring	Leaf	Boil	Food coloring	Medicine, veterinary drug	82	3.0	1.0	1.50	0.75	9.0	3.0	74.72	HRC573
*Viola philippica* Cavanilles		Violaceae	Herb	Wild	Spring	Whole plant	Boil	Vegetable	Medicine, fodder	62	2.5	1.0	1.50	1.00	7.5	4.0	69.75	HRC593
*Perilla frutescens* var. purpurascens (Hayata) H. W. Li		Lamiaceae	Herb	Wild	Summer, fall	Leaf	Seasoning	Spice	Medicine	80	2.5	1.0	1.50	1.00	7.5	3.0	67.50	HRC887
*Achyranthes longifolia* (Makino) Makino	Ya gei	Amaranthaceae	Herb	Wild	Fall, winter	Root	Stew	Tonic	Medicine, veterinary drug, fodder	26	3.0	1.0	1.50	1.50	7.5	5.0	65.81	HRC424
*Acorus calamus* L.		Araceae	Herb	Cultivated	Whole year	Tender sprout	Seasoning	Spice	Veterinary drug	73	1.0	2.0	1.50	1.00	7.5	4.0	65.70	HRC560
*Pinus massoniana* Lamb.		Pinaceae	Tree	Wild	Spring	Pollen	Soak in boiled water	Herbal tea	Religious celebration	30	4.0	1.0	1.50	1.00	9.0	4.0	64.80	HRC51
*Citrus reticulata* Blanco		Rutaceae	Shrub	Cultivated	Fall, winter	Fruit	Eat directly	Fruit	Religious celebration	78	2.0	2.0	1.50	0.50	9.0	3.0	63.18	HRC988
*Nephrolepis cordifolia* (L.) C. Presl	Bie zhen nai	Nephrolepidaceae	Herb	Wild	Spring, summer, fall, winter	Corm	Eat directly	Fruit	Medicine	73	4.0	1.0	1.50	0.50	5.5	5.0	60.23	HRC769
*Fagopyrum dibotrys* (D. Don) H. Hara	Ya weng	Polygonaceae	Herb	Cultivated	Spring, summer	Tender sprout	Boil	Vegetable	Medicine, fodder	43	3.5	1.0	1.50	1.00	6.5	4.0	58.70	HRC935
*Acorus macrospadiceus* F. N. Wei et Y. K. Li	Xi hang	Araceae	Herb	Cultivated	Whole year	Leaf	Stew	Spice	Medicine, veterinary drug	30	2.0	2.0	1.50	1.00	7.5	4.0	54.00	HRC808
*Polygonatum cyrtonema* Hua		Asparagaceae	Herb	Wild	Whole year	Tuber	Stew	Tonic	Medicine	52	1.0	1.0	1.50	1.50	9.0	5.0	52.65	HRC611
*Rubus niveus* Thunb.	Genzai	Rosaceae	Liana	Wild	Summer	Fruit	Eat directly	Fruit	Medicine, veterinary drug	85	3.0	1.0	1.50	0.50	9.0	3.0	51.64	HRC548
*Lilium brownii* var. viridulum Baker	Gai	Liliaceae	Herb	Wild	Winter, spring	Bulb	Stew, stir-fry	Tonic	Medicine	30	1.0	1.0	1.50	2.50	9.0	5.0	50.63	HRC983
*Rubus alceifolius* Poiret in Lamarck	Gei se	Rosaceae	Liana	Wild	Summer, fall	Fruit	Eat directly	Fruit	Medicine	50	2.5	2.0	1.50	0.50	9.0	3.0	50.63	HRC758
*Artemisia lactiflora* Wall. ex DC.		Asteraceae	Herb	Wild	Spring	Tender sprout	Stir-fry	Vegetable	Fodder	31	2.0	2.0	1.50	1.00	6.5	4.0	48.36	HRC641
*Coriandrum sativum* L.		Apiaceae	Herb	Cultivated	Fall, winter, spring	Leaf	Seasoning	Spice	Medicine	21	2.5	2.0	1.50	1.00	10.0	3.0	47.25	HRC1157
*Ficus tikoua* Bur.	Zu meng ha	Moraceae	Liana	Wild	Fall	Fruit	Eat directly	Fruit	Medicine	38	3.0	2.0	1.50	0.50	9.0	3.0	46.17	HRC498
*Chrysanthemum indicum* Linnaeus		Asteraceae	Herb	Wild	Spring	Tender sprout	Stir-fry	Vegetable	Medicine	20	2.0	2.0	1.50	1.00	7.5	5.0	45.00	HRC735
*Youngia japonica* (L.) DC.		Asteraceae	Herb	Wild	Spring, summer	Whole plant	Boil	Vegetable	Fodder	53	2.5	1.0	1.50	1.00	7.5	3.0	44.72	HRC960
*Pyracantha fortuneana* (Maximowicz) H. L. Li		Rosaceae	Shrub	Wild	Fall	Fruit	Eat directly	Fruit	Medicine	120	3.0	1.0	1.50	0.50	5.5	3.0	44.55	HRC215
*Setaria italica* var. germanica (Mill.) Schred.		Poaceae	Herb	Cultivated	Fall	Seed	Boil, stir-fry	Staple food	Fodder	60	1.5	1.0	1.00	2.50	6.5	3.0	43.88	HRC340
*Dioscorea bulbifera* L.	Wei yo	Dioscoreaceae	Liana	Wild	Fall	Corm	Boil, roast	Vegetable, staple food	Medicine	45	2.5	1.0	1.50	2.00	6.5	2.0	43.88	HRC706
*Cyclocodon lancifolius* (Roxburgh) Kurz		Campanulaceae	Herb	Wild	Fall	Fruit	Eat directly	Fruit		65	1.5	2.0	1.50	0.50	7.5	4.0	43.88	HRC990
*Urena lobata* Linnaeus		Malvaceae	Shrub	Wild	Fall, winter	Seed	Boil	Staple food	Medicine	37	4.0	1.0	1.50	1.00	6.5	3.0	43.29	HRC870
*Phytolacca americana* Linnaeus	Ya lai guo	Phytolaccaceae	Herb	Wild	Summer, fall	Fruit, tuberous root	Infuse with liquor, stew	Tonic	Medicine	25	1.5	1.0	3.00	1.00	7.5	5.0	42.19	HRC682
*Rubus parvifolius* Linnaeus	Ging gei	Rosaceae	Liana	Wild	Summer	Fruit	Eat directly	Fruit	Medicine	82	3.0	1.0	1.50	0.50	7.5	3.0	41.51	HRC104
*Smallanthus sonchifolius* (Poepp.) H.Rob.		Asteraceae	Herb	Cultivated	Fall, winter	Tuber	Eat directly	Fruit		81	1.5	2.0	1.50	0.50	7.5	3.0	41.01	HRC1086
*Melastoma malabathricum* Linnaeus	阿卡超拉	Melastomataceae	Shrub	Wild	Fall	Fruit	Eat directly	Fruit	Medicine, veterinary drug	91	3.0	1.0	1.50	0.50	6.5	3.0	39.93	HRC493
*Artemisia argyi* Lévl. et Van.	Wo ho	Asteraceae	Herb	Wild	Spring	Tender sprout	Stir-fry	Vegetable	Medicine, fodder	21	2.0	2.0	1.50	1.00	7.5	4.0	37.80	HRC848
*Ginkgo biloba* L.		Ginkgoaceae	Tree	Cultivated	Fall	Seed	Stew	Tonic		31	1.5	1.0	1.50	1.50	9.0	4.0	37.67	HRC1482
*Diospyros kaki* Thunb.		Ebenaceae	Tree	Cultivated	Fall, winter	Fruit	Eat directly	Fruit		95	2.0	2.0	1.50	0.50	6.5	2.0	37.05	HRC1481
*Angelica decursiva* (Miquel) Franchet & Savatier		Apiaceae	Herb	Wild	Spring	Leaf	Boil	Vegetable	Medicine	20	2.0	2.0	1.50	1.00	7.5	4.0	36.00	HRC911
*Lilium brownii* F. E. Brown ex Miellez		Liliaceae	Herb	Wild	Fall, winter	Bulb	Stir-fry, stew	Vegetable		25	1.0	1.0	1.50	2.50	7.5	5.0	35.16	HRC576
*Saurauia thyrsiflora* C. F. Liang et Y. S. Wang		Actinidiaceae	Tree	Wild	Summer	Fruit	Eat directly	Fruit	Fodder	86	2.0	1.0	1.50	0.50	9.0	3.0	34.83	HRC961
*Pleioblastus amarus* (Keng) Keng f.	A zei	Poaceae	Herb	Cultivated	Spring	Stem	Stir-fry	Vegetable	Religious celebration	68	1.5	1.0	1.50	1.00	7.5	3.0	34.43	HRC1115
*Polygonatum kingianum* Collett & Hemsley		Asparagaceae	Herb	Wild	Fall, winter	Tuber	Stew	Tonic	Medicine	40	1.0	1.0	1.50	1.50	7.5	5.0	33.75	HRC475
*Rubus lambertianus* Seringe in Candolle		Rosaceae	Liana	Wild	Fall	Fruit	Eat directly	Fruit	Medicine	76	3.0	1.0	1.50	0.50	6.5	3.0	33.35	HRC214
*Maclura cochinchinensis* (Loureiro) Corner	Li bu ru	Moraceae	Shrub	Wild	Fall	Fruit	Eat directly	Fruit	Medicine	58	2.5	1.0	1.50	0.50	7.5	4.0	32.63	HRC68
*Cardamine occulta* O.E.Schulz		Brassicaceae	Herb	Wild	Spring	Whole plant	Boil, stir-fry	Vegetable	Fodder	25	1.5	1.0	1.50	2.50	7.5	3.0	31.64	HRC974
*Zanthoxylum bungeanum* Maxim.		Rutaceae	Shrub	Cultivated	Fall	Fruit	Seasoning	Spice		38	1.0	1.0	1.50	1.00	10.0	5.0	28.50	HRC1491
*Aralia chinensis* L.		Araliaceae	Shrub	Wild	Spring	Tender sprout	Stir-fry	Vegetable	Medicine	34	2.0	1.0	1.50	1.00	9.0	3.0	27.54	HRC1475
*Sanicula orthacantha* S. Moore	Ya dou wu	Apiaceae	Herb	Wild	Spring, summer	Tender sprout	Stir-fry	Vegetable		30	1.0	2.0	1.50	1.00	7.5	4.0	27.00	HRC992
*Phytolacca acinosa* Roxburgh	Zu ziong	Phytolaccaceae	Herb	Wild	Whole year	Tuberous root	Stew	Tonic	Medicine, fodder	20	1.5	1.0	1.50	1.50	7.5	5.0	25.31	HRC830
*Citrus reticulata* ‘Shatang’		Rutaceae	Tree	Cultivated	Winter, spring	Fruit	Eat directly	Fruit		31	2.0	2.0	1.50	0.50	9.0	3.0	25.11	HRC1084
*Saurauia tristyla* DC.		Actinidiaceae	Tree	Wild	Summer	Fruit	Eat directly	Fruit		80	1.5	1.0	1.50	0.50	9.0	3.0	24.30	HRC1490
*Asarum caudigerum* Hance		Aristolochiaceae	Herb	Wild	Whole year	Whole plant	Boil, stew	Tonic	Medicine	15	2.0	1.0	1.50	2.00	6.5	4.0	23.40	HRC526
*Hellenia speciosa* (J.Koenig) S.R.Dutta	Gu shou	Costaceae	Herb	Wild	Fall, winter	Tuber	Stew	Tonic	Medicine	12	2.0	2.0	1.50	1.00	6.5	5.0	23.40	HRC692
*Stellaria media* (L.) Villars	Ya zhu zou	Caryophyllaceae	Herb	Wild	Spring	Tender sprout	Boil	Vegetable	Fodder	25	3.0	1.0	1.50	1.00	6.5	3.0	21.94	HRC995
*Asparagus lycopodineus* (Baker) Wang et Tang	七姐妹	Asparagaceae	Liana	Wild	Whole year	Tuberous root	Boil	Tonic	Medicine	22	2.0	1.0	1.50	1.00	6.5	5.0	21.45	HRC806
*Boehmeria nivea* (L.) Gaudich.	wo gu	Urticaceae	Shrub	Cultivated	Whole year	Leaf, root	Boil	Staple food	Medicine, fodder	12	3.0	1.0	3.00	1.00	6.5	3.0	21.06	HRC1030
*Zingiber mioga* (Thunb.) Rosc.		Zingiberaceae	Herb	Cultivated	Summer	Young inflorescence	Stir-fry	Vegetable		12	1.0	1.0	3.75	1.50	7.5	4.0	20.25	HRC890
*Asparagus cochinchinensis* (Lour.) Merr.	Qi jie mei	Asparagaceae	Herb	Wild	Fall, winter	Tuberous root	Boil	Tonic	Medicine	18	2.0	1.0	1.50	1.00	7.5	5.0	20.25	HRC144
*Citrus limonia* Osb.		Rutaceae	Tree	Cultivated	Fall	Fruit	Eat directly	Fruit	Veterinary drug	34	2.0	2.0	1.50	0.50	6.5	3.0	19.89	HRC798
*Melastoma dodecandrum* Lour.		Melastomataceae	Herb	Wild	Summer, fall	Fruit	Eat directly	Fruit	Medicine, dye, fodder	62	2.0	1.0	1.50	0.50	6.5	3.0	18.14	HRC781
*Indigofera esquirolii* H. Leveille	dong	Fabaceae	Shrub	Wild	Fall, winter	Seed	Boil	Staple food	Medicine	37	2.5	1.0	1.00	1.00	6.5	3.0	18.04	HRC101
*Canna indica* L.		Cannaceae	Herb	Cultivated	Whole year	Whole plant	Stew	Tonic	Medicine, fodder	16	2.0	1.0	1.50	1.00	7.5	5.0	18.00	HRC788
*Pyrus calleryana* Dcne.		Rosaceae	Tree	Wild	Fall	Fruit	Eat directly	Fruit		73	2.5	1.0	1.50	0.50	4.0	3.0	16.43	HRC982
*Angiopteris fokiensis* Hieron.		Marattiaceae	Herb	Wild	Whole year	Stem	Boil	Tonic	Medicine	12	2.0	1.0	1.50	1.00	9.0	5.0	16.20	HRC926
*Lithocarpus litseifolius* (Hance) Chun		Fagaceae	Tree	Cultivated	Whole year	Leaf	Boil	Herbal tea		16	0.5	2.0	2.00	1.00	10.0	5.0	16.00	HRC443
*Morus australis* Poir.		Moraceae	Shrub	Cultivated	Spring, fall	Leaf, fruit	Boil, eat directly	Vegetable, fruit		18	1.0	1.0	3.00	1.50	6.5	3.0	15.80	HRC1078
*Diospyros kaki* var. silvestris Makino		Ebenaceae	Tree	Wild	Fall, winter	Fruit	Eat directly	Fruit		54	1.5	2.0	1.50	0.50	6.5	2.0	15.80	HRC100
*Indigofera bungeana* Walpers		Fabaceae	Shrub	Wild	Fall, winter	Seed	Boil	Staple food	Medicine	31	2.5	1.0	1.00	1.00	6.5	3.0	15.11	HRC860
*Rubus corchorifolius* L. f.	Gei jia zhu	Rosaceae	Shrub	Wild	Summer	Fruit	Eat directly	Fruit		36	1.5	1.0	1.50	0.50	10.0	3.0	12.15	HRC990
*Kadsura longipedunculata* Finet & Gagnepain		Schisandraceae	Shrub	Wild	Fall	Fruit	Eat directly	Fruit	Medicine	38	1.0	1.0	1.50	0.50	9.0	4.0	10.26	HRC143
*Ligustrum lucidum* W. T. Aiton		Oleaceae	Tree	Cultivated	Fall, winter	Fruit	Infuse with liquor	Tonic	Medicine	22	1.5	1.0	1.50	0.75	5.5	5.0	10.21	HRC1485
*Dahlia pinnata* Cav.		Asteraceae	Herb	Cultivated	Whole year	Tuber	Stew	Tonic	Medicine	12	1.0	1.0	1.50	1.50	6.5	5.0	8.78	HRC694
*Zingiber striolatum* Diels		Zingiberaceae	Herb	Wild	Spring	Tender sprout	Stir-fry	Vegetable	Medicine	16	1.0	1.0	1.50	1.00	9.0	3.0	6.48	HRC890
*Commelina benghalensis* Linnaeus	Wo zou wu	Commelinaceae	Herb	Wild	Spring, summer	Tender sprout	Boil	Vegetable	Fodder	16	2.0	1.0	1.50	1.00	6.5	2.0	6.24	HRC822
*Prunus mume* Siebold & Zucc.	Bi ma	Rosaceae	Tree	Cultivated	Summer	Fruit	Eat directly	Fruit	Landscaping	35	1.0	1.0	1.50	0.50	7.5	3.0	5.91	HRC1170
*Solanum lyratum* Thunberg in Murray	Ya gei	Solanaceae	Herb	Wild	Fall	Fruit	Eat directly	Fruit	Medicine	20	1.5	1.0	1.50	0.50	7.5	3.0	5.06	HRC710
*Ficus erecta* Thunb		Moraceae	Shrub	Wild	Summer	Fruit	Eat directly	Fruit		22	1.5	1.0	1.50	0.50	6.5	3.0	4.83	HRC99
*Punica granatum* L.		Lythraceae	Tree	Cultivated	Fall, winter	Fruit	Eat directly	Fruit		32	1.0	1.0	1.50	0.50	6.5	3.0	4.68	HRC1037
*Lindera pulcherrima* var. attenuata Allen		Lauraceae	Tree	Wild	Whole year	Leaf	Sun-dry, grind to powder, then boil	Staple food		3	4.0	0.8	1.50	1.50	5.5	3.0	3.56	HRC1232
*Ulmus castaneifolia* Hemsl	Ya hui	Ulmaceae	Tree	Wild	Whole year	Bark	Sun-dry, grind to powder, then boil	Staple food		5	2.0	0.8	1.00	1.50	6.5	3.0	2.34	HRC1001
*Duhaldea cappa* Pruski & Anderberg		Asteraceae	Herb	Wild	Whole year	Whole plant	Boil	Lye water	Medicine	9	1.0	1.0	1.50	0.75	7.5	2.0	1.52	HRC874
*Lysimachia christiniae* Hance		Primulaceae	Herb	Wild	Spring	Tender sprout	Boil, stir-fry	Vegetable	Medicine	12	1.0	0.5	1.50	2.50	6.5	1.0	1.46	HRC793

**Table 3:** Statistical classification of edible plants used by the Baiku Yao people

Type	Quantity	Proportion of total families (genera) (%)	Number of species	Proportion of total species (%)
Multifamily (≥ 5 species)	14	20.59	114	58.46
Oligofamily (2–4 species)	18	26.47	45	23.08
Monofamily (1 species)	36	52.94	36	18.46
Total (Families)	68	100.00	195	100.00
Multigenus (≥ 5 species)	3	2.11	17	8.72
Oligogenus (2–4 species)	28	19.72	67	34.36
Monogenus (1 species)	111	78.17	111	56.92
Total (Genera)	142	100.00	195	100.00

The selection of edible plants by the Baiku Yao demonstrates a preference for specific families with a wide variety of plant species. The Asteraceae (18 species), Rosaceae (14 species), and Fabaceae (10 species) have the highest number of edible plant species, reflecting their significant role in the traditional dietary culture of the Baiku Yao. The Rosaceae family, with its diverse fruit offerings such as apples, pears, and peaches, caters extensively to the daily nutritional needs and snack preferences of the Baiku Yao community. These fruits are crucial for providing essential vitamins, minerals, and antioxidants that contribute significantly to the overall health of the community. On the other hand, plants from the Asteraceae and Fabaceae families primarily contribute a variety of vegetables, enriching the Baiku Yao’s diet with a range of leafy greens and legumes. These vegetables are not only fundamental to their everyday meals but also offer a variety of nutrients important for maintaining a balanced diet.

At the genera level, the selection of edible plants shows significant diversity. Prominently, *Dioscorea* (6 species), *Rubus* (6 species), and *Allium* (5 species) are among the most represented, each playing a distinct role in the Baiku Yao’s diet. *Dioscorea* serves as an important source of carbohydrates for the local community; *Rubus* offers fruits, which are crucial for their nutritional content and flavor. *Allium*, with five species, can be utilized both as a vital seasoning and as a vegetable, enhancing meals with both taste and nutritional benefits.

### Life forms and resource types

**Fig. 2:**
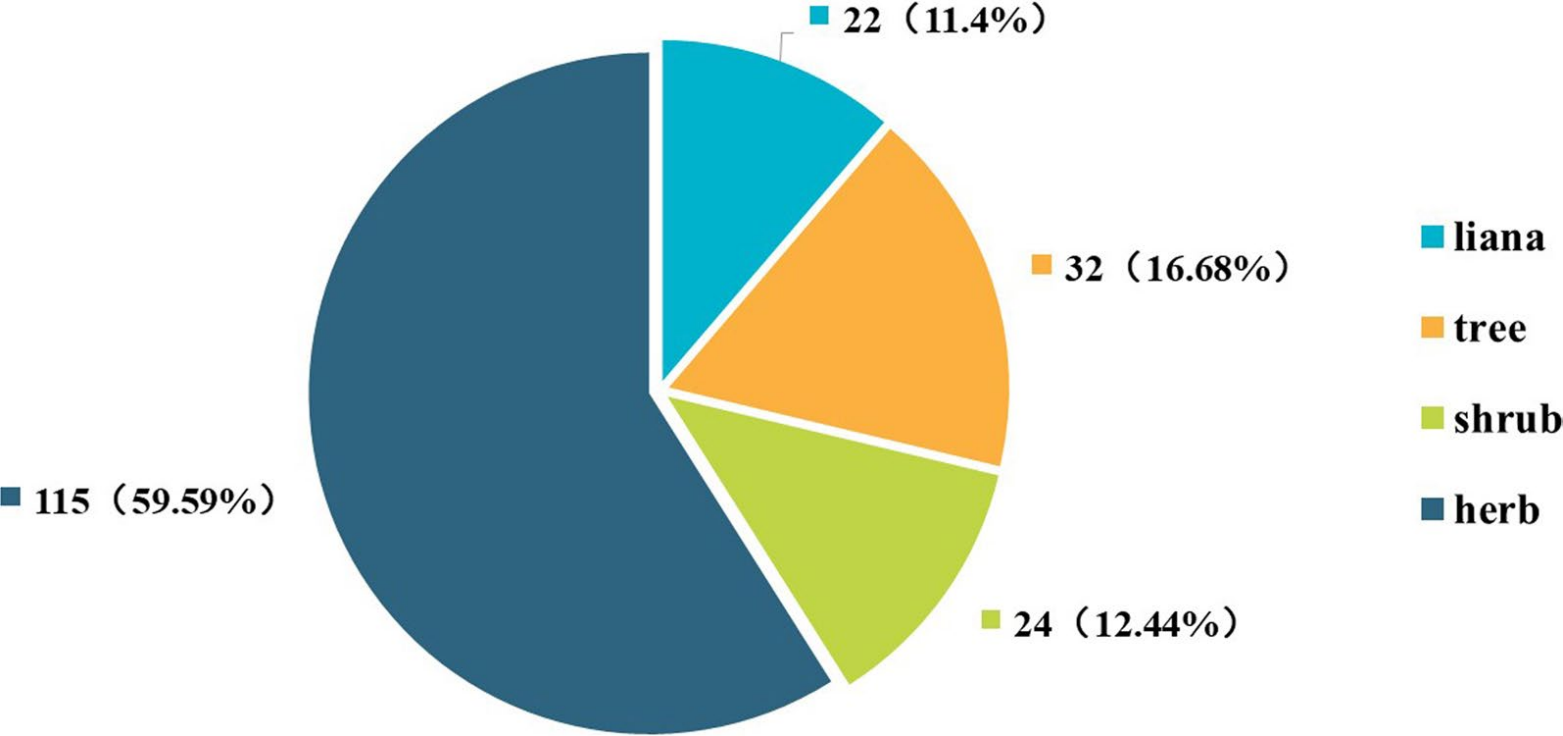
Life forms of wild edible plants used by the Baiku Yao

Regarding the life forms of Baiku Yao edible plants, herbaceous plants, numbering 115 species, account for 58.97%, making them the most common life form (Fig. 2). This prevalence may be linked to their larger biomass and greater accessibility. Tree species, totaling 34, comprise 17.44%, representing the second most common life form, followed by shrubs and lianas, with 24 and 22 species making up 12.31% and 11.28%, respectively. This distribution likely reflects the ecological characteristics of the Baiku Yao region, where herbaceous plants dominate and are more readily available, and their utility in the Baiku Yao diet.

The study result reflects the community’s use of various life forms to adapt to their specific environmental and cultural needs. Herbaceous plants are particularly common in this mountainous region due to their rapid growth and reproductive capabilities, which suit the dynamic changes and irregular agricultural activities of the area. These plants are easy to gather and cultivate, making them a crucial part of the daily diet, especially when providing seasonal vegetables and medicinal resources. Trees and shrubs, on the other hand, provide more stable and long-term resources, which are essential for ensuring a year-round food supply.

Regarding resource types, wild resources dominate with 103 species, slightly over 50%, while cultivated plants comprise 89 species, close to half (approximately 46%). The resource type indicates that the Baiku Yao does not exhibit a marked preference for either wild or cultivated sources, relying nearly equally on both. *Mentha canadensis*(mint), *Eriobotrya japonica*, and *Broussonetia papyrifera* are cultivated and can also be collected in the wild. Mint is a commonly used seasoning in Baiku Yao cuisine, *E. japonica* is a favored fruit, and *B. papyrifera* is not frequently consumed; it serves as both food and an excellent fodder source. Its high protein content in leaves, identified as a high-quality new feed, necessitates cultivation alongside wild harvesting [19]. Overall, the Baiku Yao’s reliance on both wild and cultivated resources is almost equal, reflecting a wide variety of wild edible plants and diverse cultivated species. This phenomenon demonstrates the Baiku Yao community’s profound survival wisdom in the mountainous and land-scarce areas of the Yunnan–Guizhou Plateau. With limited arable land and distinct seasonal changes, large-scale agricultural production is not feasible. Thus, they adopt diverse food procurement strategies, relying heavily on wild edible plants and practicing mixed cropping on limited land to mitigate potential food crises from sudden disasters, which is most notably evident in their management of homegarden plants [20].

### Edible parts

**Fig. 3:**
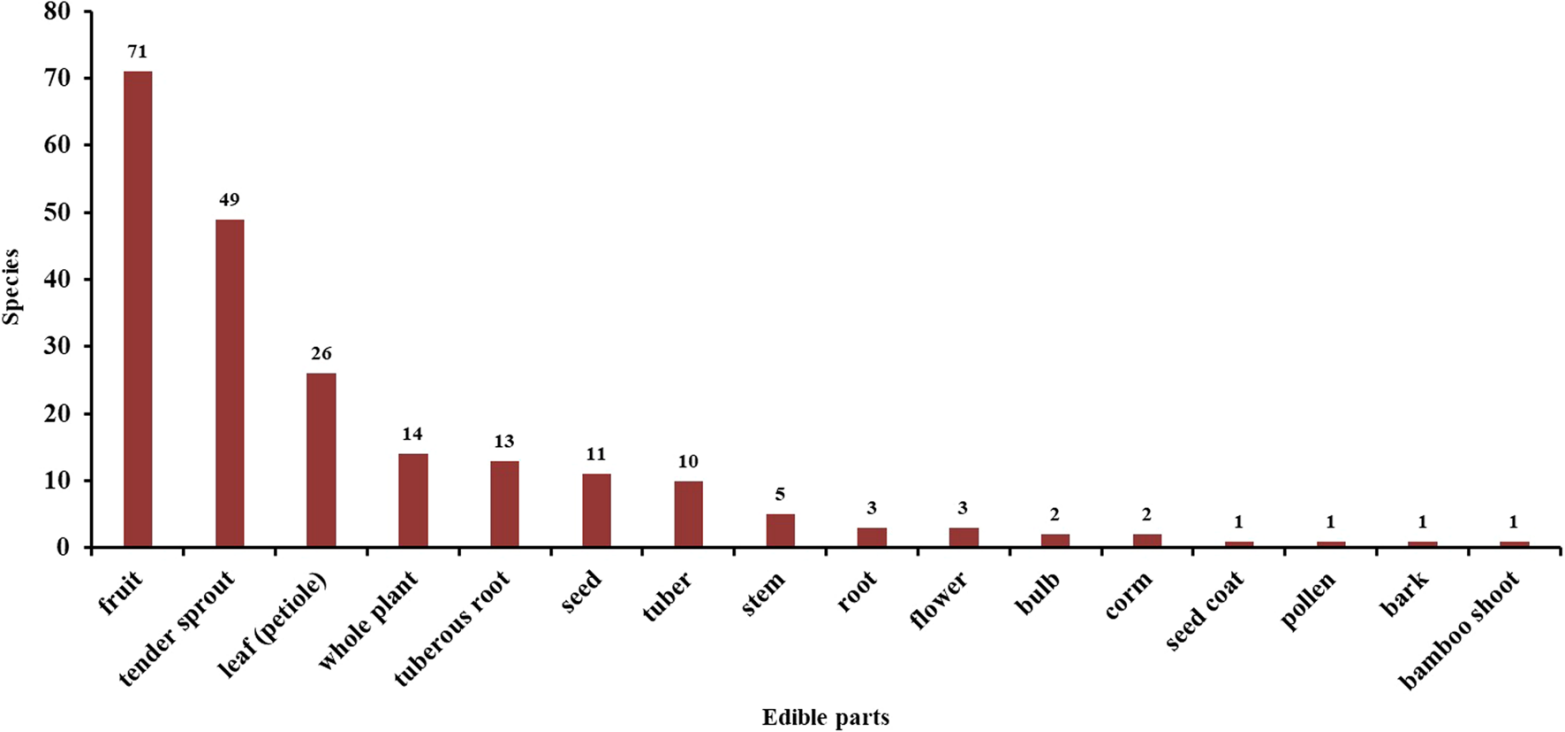
Used parts of edible plants by Baiku Yao people

The Baiku Yao community utilizes a wide array of species and parts from wild edible plants. This study categorizes the edible parts as follows: fruit, tender sprout, leaf (petiole), whole plant, tuberous root, seed, tuber, stem, root, flower (young inflorescence), bulb, corm, seed coat, pollen, bark, bamboo shoot (Fig. 3). The data show that the most numerous are plants with edible fruits, totaling 74 species. This is likely due to the high sugar content and pleasant taste of fruits. Following fruits, tender sprouts, and leaves represent the second and third most common categories, with 49 species and 26 species, respectively, which are highly edible and the main source of wild vegetables.

The data further include 14 species with edible whole plants, 13 with tuberous roots, 11 with seeds, and 10 with tubers. Lesser encountered categories are stems with 5 species, roots and flowers (young inflorescence), each with 3 species, and bulb, corm, seed coat, pollen, bark, and bamboo shoot, each with 1 species, bringing the total count to 213. Despite their lower numbers, the culinary and nutritional value of less common parts like flowers should not be underestimated; for instance, using *Buddleja officinalis* flowers in dishes is quite common locally. Additionally, 18 plant species were found to have more than two edible parts, such as the *Hylocereus undatus* with edible flowers and fruits, *Campanumoea javanica* with edible tubers and fruits, and *Zingiber mioga* with edible whole plants and tender inflorescences. The multiple edible parts of certain species provide a reliable food source throughout different times of the year. This not only helps keep the community well-fed by offering a variety of nutrients but also shows deep local knowledge about how to use nature wisely and sustainably.

The wide variety of edible plant types and parts used by Baiku Yao offers a comprehensive and ample supply of nutrients for the local diet, including proteins, fats, carbohydrates, vitamins, and minerals. Ensuring a balanced dietary structure and nutrition is crucial for maintaining health.

### Harvesting and processing methods

In our in-depth study of the Baiku Yao’s traditional harvesting habits, we observed a clear seasonality in their selection of wild edible plants. Spring and autumn, when most plants are in their peak growth phase, are the periods of most frequent harvesting activities. During these two seasons, the plant species harvested account for 56.39% of the total species surveyed. This seasonal collection not only reflects the close relationship between plant growth cycles and harvesting activities but also highlights the richness of edible parts available in specific seasons. For instance, spring is the growth period for many plants’ tender leaves and flowers, while autumn is the time for fruit and seed maturation.

Although the variety of plants harvested in winter decreases to 49 species (17.38% of the total), the activity continues, mainly focusing on rhizomes and some evergreens that survive low temperatures. The variety further reduces to 33 species (11.70%) in summer, possibly due to the vigorous growth of plants, leafy plants age and become less palatable, and fruit plants are not yet mature, leading to reduced harvesting in summer. Notably, 41 species can be harvested year-round, accounting for 14.54%, reflecting the Baiku Yao’s continuous dependence on these multi-seasonal plants.

**Fig. 4:**
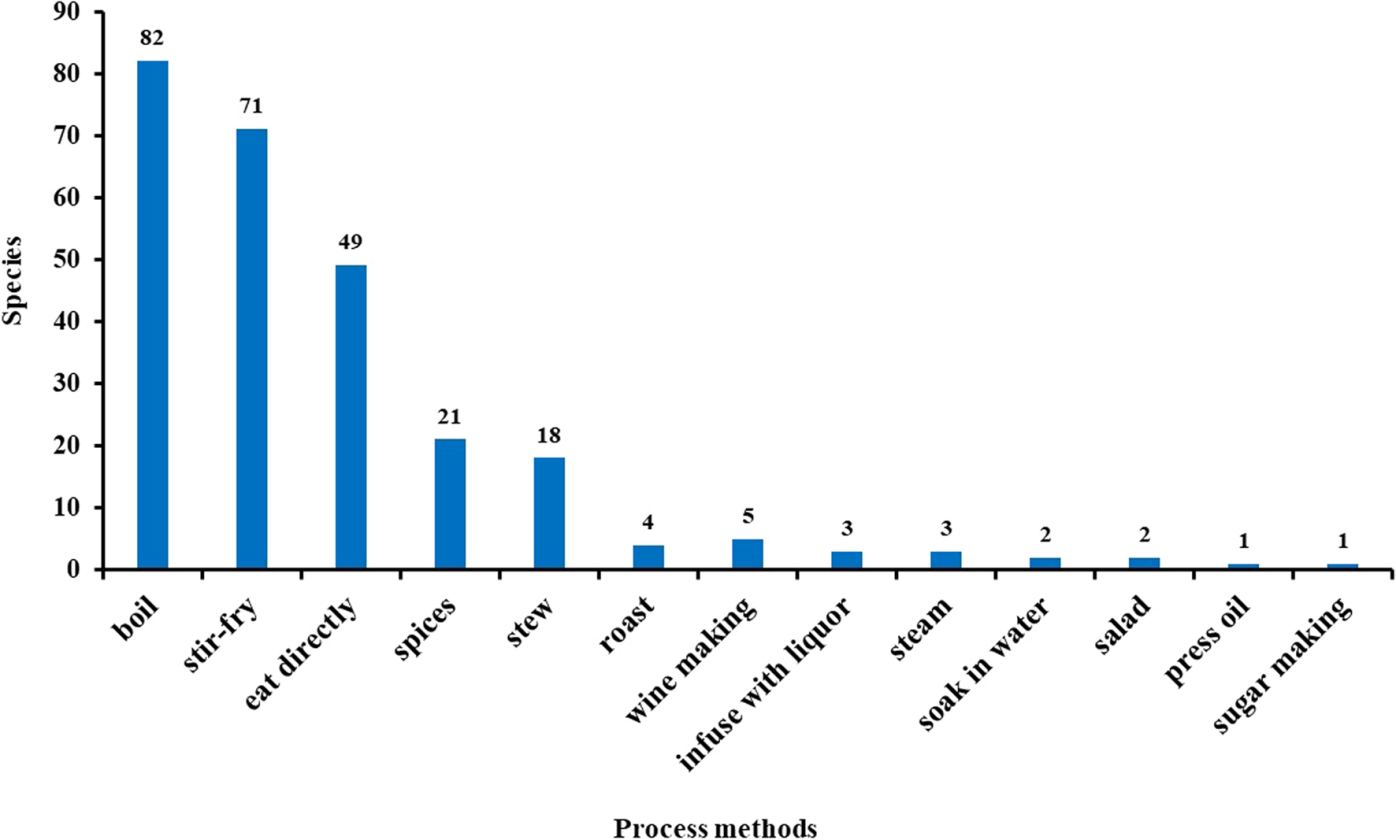
Processing methods of food plants by Baiku Yao people

The Baiku Yao also exhibit significant diversity in their processing and utilization methods of wild edible plants, which are closely linked to their living environment and self-sufficient lifestyle. Statistical analysis (see Fig. 4) shows that the primary processing methods include boiling, simple stir-frying, and raw consumption. The Baiku Yao people believe that boiling can reduce the loss of nutrients in food while aiding in the body’s absorption. Therefore, they have a strong preference for boiling in food processing. However, as their contact and exchange with the outside world increase, stir-frying has become increasingly favored by the Baiku Yao, which can further enrich food flavor.

Nearly one-fifth of the plants are used as seasoning or spices, possibly involving roots, stems, or mature seeds harvested in specific seasons. Due to their long-standing location in inland mountainous regions where salt is scarce, the Baiku Yao has developed many plant species for seasoning to enhance the flavor of their food. The processing methods of seasonings are diverse; they can be stewed, boiled, or stir-fried together with meat, directly combined with salt and oil to form dipping sauces, or used in the initial meat marination for stewing or stir-frying. To avoid excessive repetition in this study, the term "seasonings" is uniformly used to categorize the related plant processing types. Other methods like roasting, steaming, and making cold dishes (salad) are less common but demonstrate the unique utilization of different plant parts. Additionally, a few plants undergo deep processing, such as extraction of sugars or oils, typically involving mature parts harvested in specific seasons.

These findings not only reveal the Baiku Yao’s extensive knowledge in harvesting and processing wild plants but also show how they combine the seasonal characteristics of plants with the utilization of different parts to adapt to their living environment and sustainable development needs. This accumulated experience is closely linked to their history of living in relatively isolated mountainous areas and relying on a self-sufficient lifestyle.

### Application categories

**Fig. 5:**
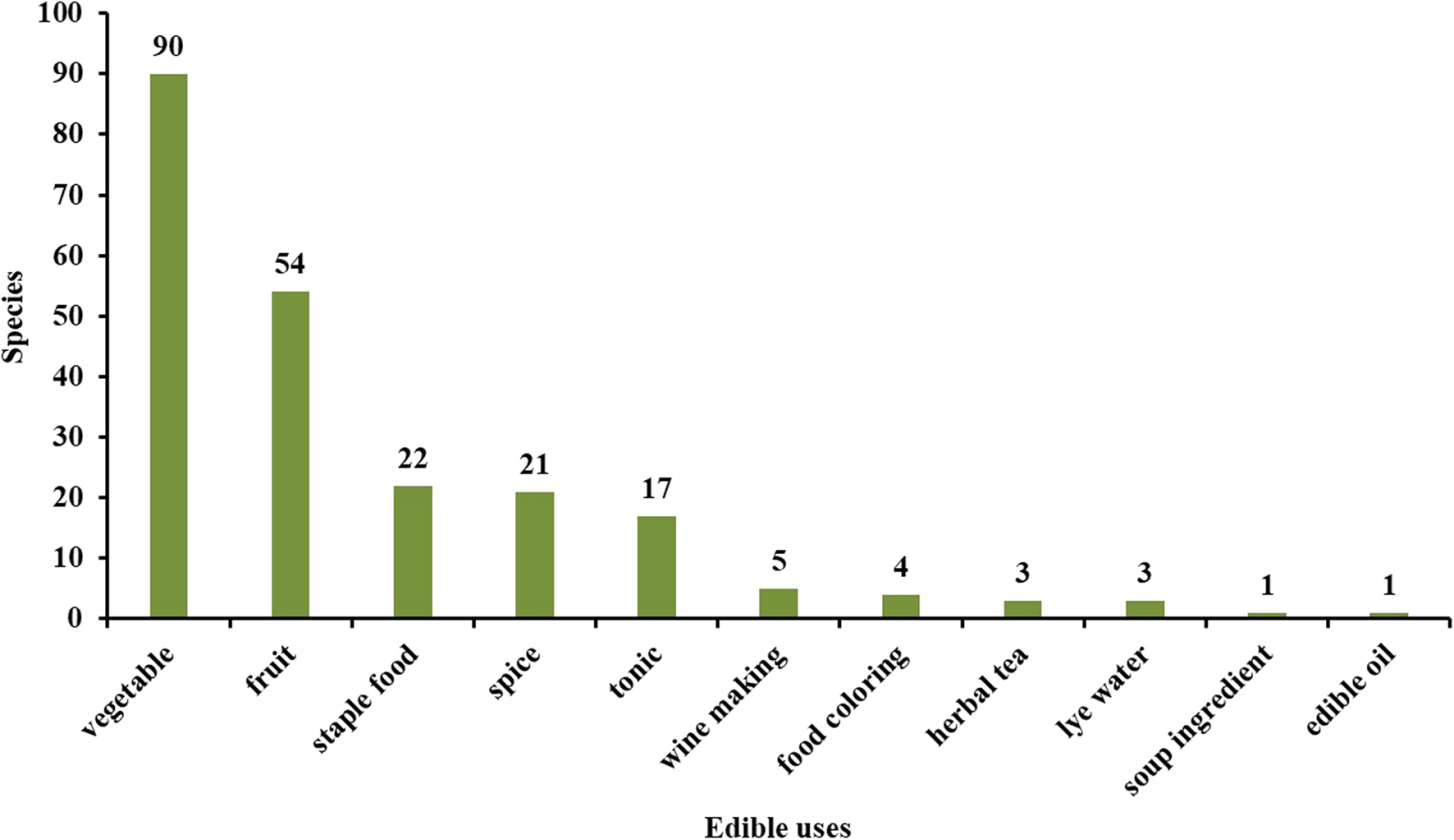
Use purpose of food plants by Baiku Yao people

Our study reveals the Baiku Yao’s comprehensive use of wild plants in their traditional dietary practices (Fig. 5), an approach of significant research value in ecology and cultural anthropology. With 90 varieties, vegetables lead the plant utilization categories, reflecting the Baiku Yao’s extensive development and application of wild plant resources in their long-term adaptation to the local environment. These vegetables not only provide the Baiku Yao with essential daily nutrition but also play a crucial role in maintaining biodiversity and food security.

The Baiku Yao community consumes 54 different fruit species. These fruit species not only enrich the diet in taste and nutrition but may also serve as a buffer in the seasonal food supply. The utilization of 21 spice plants illustrates the complexity of Baiku Yao cuisine and its refined pursuit of flavors. Additionally, given that their diet primarily comprises light foods with minimal oil and salt, the rich variety of spice plants enhances flavor and nutrition. Using 22 staple food plants, such as various tubers and grains, underscores the diversity of carbohydrate sources in the Baiku Yao diet.

The 17 recorded species for nutritional tonics often contain significant medicinal value, which is crucial in traditional medical practices. The Baiku Yao’s knowledge and use of these plants reflect their deep understanding and intergenerational transmission of local plant medicinal properties. Moreover, other categories like brewing plants, edible pigments, and tea substitutes, though numerically fewer, are vital expressions of Baiku Yao’s cultural characteristics. Five recorded brewing plants reveal the role of brewing techniques in social ceremonies and daily life. Four edible pigment plants demonstrate the Baiku Yao’s aesthetic pursuit in visual enhancement and food processing. Additionally, a food additive species is usually burnt into ashes and added to wrapping zongzi (rice dumplings), enhancing this traditional dish’s taste and preservation.

**Fig. 6:**
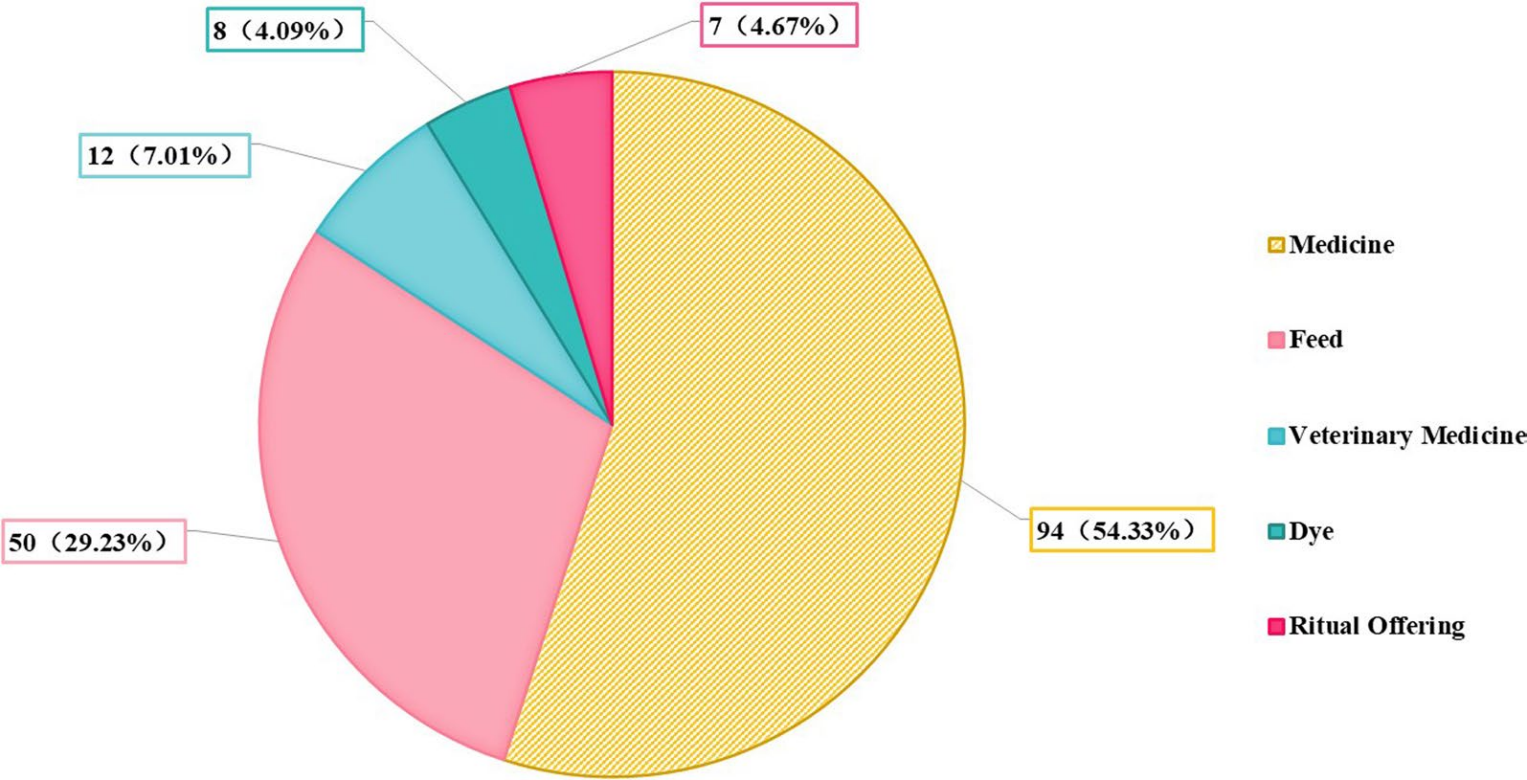
Multi-functional food plants used by Baiku Yao people

Statistical data show that the Baiku Yao utilize 195 plant species to meet their dietary needs, with 124 having additional uses beyond essential food functions (Fig. 6). The multifunctional use of these plants illustrates the Baiku Yao’s exceptional wisdom in traditional knowledge and biodiversity utilization. Specifically, 94 species also serve medicinal purposes, confirming the deep-rooted culture of food therapy among the Baiku Yao and reflecting their profound legacy in local medical knowledge. The use of 50 plants as fodder highlights the Baiku Yao’s adaptability and resource recycling in agriculture and animal husbandry. The documentation of 12 veterinary plants reveals traditional methods of animal health management, potentially providing a foundational basis for modern veterinary science. The utilization of 7 dye plants and 8 ceremonial plants profoundly demonstrates the multi-dimensional role of plants in the Baiku Yao’s daily life and spiritual culture.

The versatile use of these plants not only adds to the Baiku Yao’s cultural wealth but also tangibly reflects their spiritual beliefs. The Baiku Yao’s comprehensive use of plants demonstrates a thorough ecological knowledge and a dedication to living in harmony with both nature and their community. This profound connection with the environment offers vital insights into preserving their distinctive cultural identity.

### CFSI indices

Significant variations are evident in the cultural food significance index (CFSI) across different species. As per the study by Pieroni et al. (2001), wild edible plants are categorized into six levels based on their CFSI values: very high (CFSI = 1000 and above), high (CFSI = 999–500), moderate (CFSI = 499–100), low (CFSI = 99–50), very low (CFSI = 50–10), and negligible (CFSI < 10). There is a notable disparity in the distribution of plants across these categories. Specifically, the moderate category comprises 67 plant species, followed by the very low category with 51 species, and then the low (29 species), high (14 species), very high (23 species), and negligible (11 species) categories [15].

In studying the wild edible plants of the Baiku Yao region, special attention was paid to their cultural food significance index (CFSI) to assess the importance of different plants in the lives of local people. In this assessment, 23 plant species were assigned significantly high importance (CFSI = 1000 and above). These include *Ipomoea batatas* (sweet potatoes), *Zanthoxylum armatum*, *Glycine max* (soybeans), *Morus alba*, *Raphanus sativus* (radish), *Musa basjoo*, *Zingiber officinale* (ginger), *Cucumis sativus* (cucumber), *Pisum sativum* (peas), *Capsicum annuum* (chili peppers), *Allium hookeri*, *Plantago asiatica*, etc. These plants are typically common in daily diets, with a characteristic presence of spice and side-dish plants, and most are perennial herbs that can be harvested throughout the year. Their edible parts are diverse, including tubers, rhizomes, leaves, fruits, seeds, and other parts of the herbaceous plants.

Among all these plants, sweet potato has the most prominent CFSI value. As an important source of energy and protein nutrition for local residents, it is not only easy to cultivate but also has multiple usable parts and a high frequency of consumption. It also has various processing and culinary methods, hence its high CFSI value. Following this is *Zanthoxylum armatum*, which also has a high CFSI value and is typically used as a spice stewed with meat to eliminate the gamey taste. Plants like ginger, chili peppers, perilla, garlic, and onions also play a significant role in seasoning. Particularly noteworthy is *Houttuynia cordata*, also known as "yuxingcao," the wild vegetable with an extremely high CFSI. *H. cordata* is not only edible but also has medicinal value. It is processed locally in various ways, including stir-frying and cold salads. Rich in protein, minerals, crude fiber, and various vitamins, it contains active components like houttuynine and decanoyl acetaldehyde, offering anti-inflammatory, antibacterial, antioxidant, and antiviral properties [21, 22]. It is widely used in the treatment of respiratory diseases, urological diseases, digestive diseases, dermatological conditions, and gynecological ailments [23]. The culinary methods for *H. cordata* are versatile; it can be washed, roots plucked, and mixed with salt, sugar, vinegar, etc., for cold salad consumption. During the summer and autumn, tender stems and leaves are picked, blanched, and rinsed for stir-frying or soup preparation. In winter and spring, its rhizomes are dug up, salted, and consumed, generally preferring the light reddish-brown parts with complete stems and leaves, free of impurities. Furthermore, as a natural product rich in various nutrients and bioactive components, *H. cordata* has attributes such as safety, nutrition, and minimal side effects [24]. When added to feed, it not only enhances nutritional and medicinal values but also improves palatability, making it an ideal substitute for antibiotics and an emerging additive in animal feed [24].

In the high CFSI (CFSI = 999–500) category, there are 14 plant species, most of which are wild edible plants commonly classified as vegetables. These plants are not only valued for their nutritional content but also for their multifunctionality, making them a focal point of research. Typically processed through cooking methods such as frying or boiling, they form an indispensable part of the local diet. Notably, these plants satisfy not just dietary needs but also possess significant medicinal value, and as fodder, they supplement livestock’s food sources. This underscores the plants’ multiple roles in the ecosystem, serving both as a source of human food and as nutritional providers for animals.

The moderate CFSI (CFSI = 499–100) category includes 66 plant species, representing the largest proportion in all categories. Among these, 39 are herbaceous, mainly encompassing various fruits and vegetables. These plants hold a significant place in the daily diet of local communities and, due to their medicinal properties, are also vitally important in traditional medicine. For instance, *E. japonica* is not only widely cultivated as a food source but also valued for its medicinal properties. Similarly, plants like *B. papyrifera*, *Artemisia indica*, and *Leucocasia gigantea* exhibit their multifunctionality. Additionally, this category includes plants like *Bambusa chungii*, used for religious rituals, and *Buddleja officinalis*, known for its dyeing properties, revealing the profound influence of culture and tradition in the utilization of edible plants.

In the low CFSI (CFSI = 99–50) and very low CFSI (CFSI = 50–10) categories, there are 28 and 51 plant species, respectively. These plants are primarily medicinal herbs, covering a variety of edible categories, from vegetables and fruits to staple foods, supplements, and spices. In these categories, about 90% of the supplementary edible plants are distributed, which are not only nutritionally diverse but also hold a significant place in dietary therapy and folk medicine. Their lower CFSI values are likely due to the plants’ more limited uses and less frequent consumption as supplements. Finally, in the negligible CFSI category (< 10), there are 11 plant species. Although these plants currently hold a lower status in the prevailing food culture, their existence still has potential importance for the conservation of ecological diversity and cultural heritage.

### RFC values

Based on the data from the interviews (Table 4), we can observe that certain plants are closely linked to the livelihoods and daily lives of the local residents through the relative citation frequency (RFC) values. This table shows the top 10 edible plants most familiar within the local community, with species such as *Zingiber officinale*, *Zea mays*, and *Oryza sativa* not only ranking high in RFC values but also being commonly cultivated and consumed in the region, indicating their crucial role in local agricultural production and culinary culture.

**Table 4:** Top 10 edible plants used by the Baiku Yao by RFC ranking

No.	Species	CFSI	RFC
1	*Zingiber officinale*	2092.50	1
2	*Zea mays*	1464.75	1
3	*Oryza sativa*	523.13	1
4	*Prunus persica*	251.1	1
5	*Prunus salicina*	188.33	1
6	*Myrica rubra*	113.00	1
7	*Capsicum annuum*	1647.00	0.98
8	*Musa basjoo*	2126.00	0.97
9	*Brassica rapa* var.* oleifera*	1458.00	0.97
10	*Solanum melongena*	437.40	0.97

The FCs in the RFC calculation reflect the total number of mentions of a specific plant species across all informants, corresponding to the quotation index (QI) within the cultural food significance index (CFSI), indicating that plants with higher RFC values often possess higher CFSI scores. However, since the CFSI evaluation considers various other factors, there are instances in this table where certain plants, including *Oryza sativa* (the most common staple food), *Prunus persica*, *Prunus salicina*, and *Myrica rubra*, have high RFC values. However, their CFSI scores do not exceed 1000. This phenomenon suggests that while they may be important components of the community’s daily diet, their contribution to culinary culture is relatively small compared to other crops.

### Utilization strategies and heritage of Baiku Yao edible plants

The Baiku Yao, predominantly residing in the deep mountainous area, have sustained their millennia-old agricultural era, which continues to play a vital role today. They inherit traditional agricultural practices, farming according to the seasons, working from sunrise to sunset, and growing major crops, including corn, soybeans, rice, cotton, and oilseeds [20]. However, the scarcity of fertile land and underdeveloped transportation have compelled the Baiku Yao residents to intensify their exploration of surrounding wild edible plant resources and innovate their own cultivation systems. Firstly, their acquisition of wild edible plants encompasses not only staple plants rich in resources (such as *L. pulcherrima* var. *attenuata*, *Ulmus castaneifolia*, *Dioscorea japonica*, and *Dioscorea persimilis*) but also a variety of vegetables and wild fruits for nutritional supplementation. Secondly, they adopt diverse species cultivation management strategies to ensure necessary food sources, such as intercropping sweet potatoes, soybeans, and cannabis in cornfields and planting various edible plants in courtyards less than 10 square meters [20]. Additionally, they use aromatic plants to enhance the pleasure and flavor of their diet, and medicinal and edible plants for nutritional supplementation, thereby constructing a rich and complex food system to cope with harsh living conditions.

**Fig. 7:**
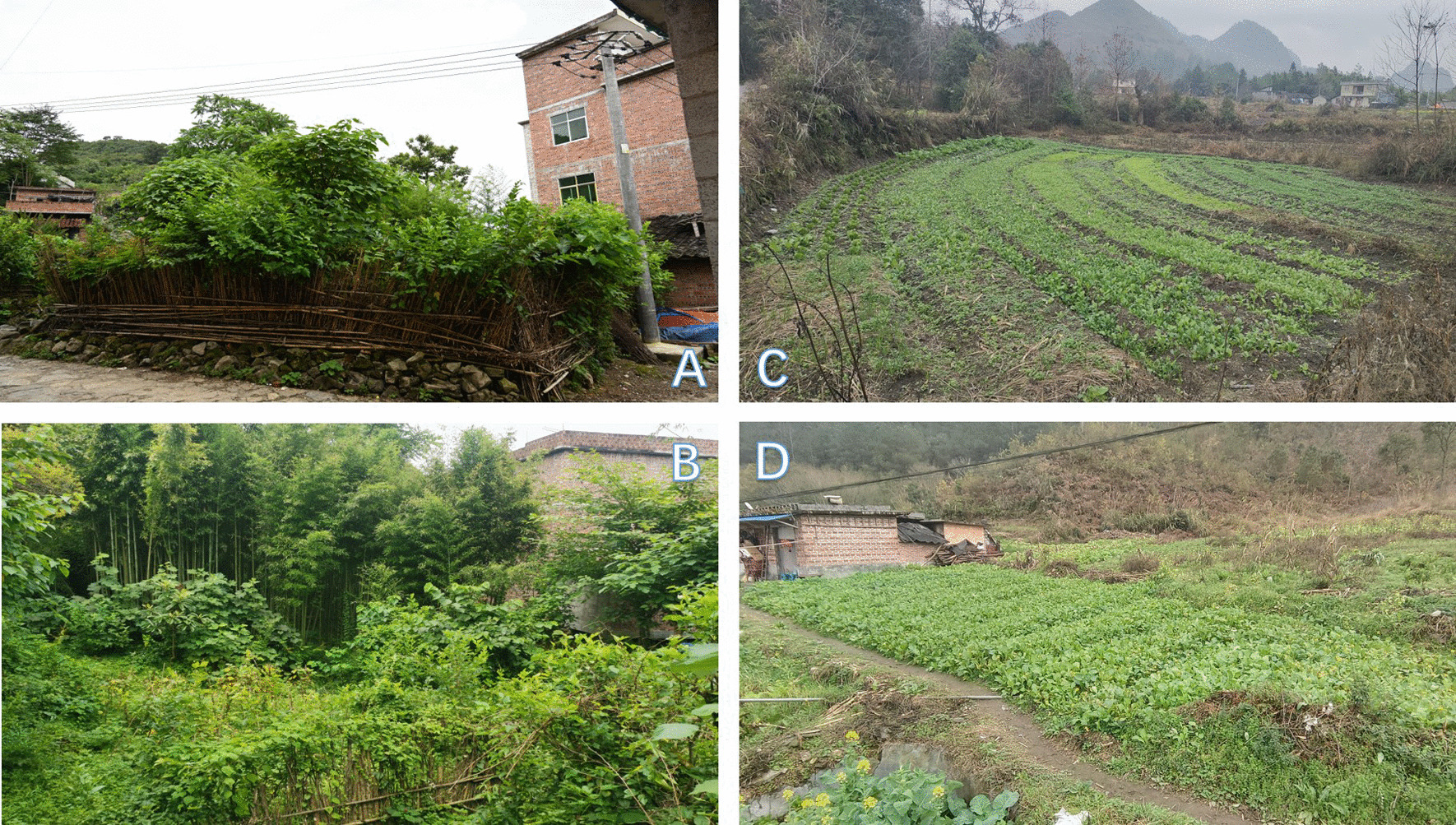
Homegardens with different strategies (**A** and **B** show the homegardens of the Baiku Yao village, while **C** and **D** show the homegardens of the Han villages around the Baiku Yao communities.)

In our survey of the Baiku Yao community, we found that their rural courtyards play a crucial role in preserving and transmitting traditional knowledge related to edible plants. Surrounding the Baiku Yao community are Zhuang and Han villages, which, compared to the Baiku Yao community, have more arable land. Due to more convenient transportation, villagers often do not need to rely on collecting wild edible plants for their daily dietary supplementation. This has led them to generally choose larger-scale crops, with relatively fewer varieties of edible plants cultivated (Fig. 7). In contrast, the Baiku Yao community, facing different geographical and economic environments with more mountains and less arable land, fully uses the limited space in their small rural courtyards to cultivate diverse edible plants. This intensive and diverse planting approach not only demonstrates the Baiku Yao community’s effective utilization of land resources but also reflects their rich experience in knowledge and utilization of edible plants [20]. However, this traditional knowledge, particularly in the Baiku Yao community, faces challenges from modernization and threats from the younger generation, who are leaving to work outside and abandoning traditional agricultural lifestyles. Therefore, it is very important to record, safeguard, and pass on this important traditional knowledge, requiring effort and support from everyone in society.

### Distinguishing features of local food plant culture

Although comprehensive ethnobotanical studies focusing on specific areas in Guangxi are relatively limited, our prior research has thoroughly explored the edible plants in the Fangchenggang area [25]. The Zhuang ethnic group predominantly inhabits this region, which is situated near the sea. It features a climate and ecological environment that distinctly contrasts with that of the inland mountainous areas. Such environmental differences directly influence the diversity of local plant species and their local folk uses.

Because of its proximity to the sea, the Fangchenggang area possesses a rich and diverse flora, which differs significantly from that of the Baiku Yao communities, which are in the mountainous region. For example, the Zhuang people in Fangchenggang can utilize a greater variety of plants adapted to the humid and hot climate, such as *Abrus precatorius*, *Pentaphragma spicatum*, and *Hedyotis effuse* [25], which are rare in the Baiku Yao area. These ecological differences lead to distinct choices in edible plants, thereby influencing food preparation and consumption habits.

Notably, many edible plants in the Zhuang community of Fangchenggang are used as tea substitutes or other beverages and are closely associated with the local humid and hot climate [25]. In high-temperature environments, people tend to choose beverages that offer a cooling sensation or have heat-relieving effects. The widespread use of tea substitute plants not only reflects adaptation to the environment but also highlights dietary preferences for refreshing drinks. Additionally, the humid and hot climate facilitates the fermentation process, and the Zhuang preference for fermented foods, especially sour-tasting ones, further aids in food preservation [25]. In contrast, the use of fermented foods is less frequent in the Baiku Yao community, likely due to its drier climate and traditional food preservation techniques.

On the other hand, geographically adjacent, Guangdong Province and Guangxi Province share certain cultural and linguistic similarities, yet they exhibit significant differences in some cultural practices. For instance, both the Cantonese and Hakka regions of Guangdong are renowned for their rich culture of herbal soups and herbal teas, influenced significantly by the open geographical location and level of economic development [26–28]. The multiple ports and developed commercial activities in Guangdong facilitate the integration of foreign cultures with local traditions, enriching the local medicinal cuisine. In contrast, the Baiku Yao area, due to its relatively isolated geographical location and underdeveloped transportation conditions, has been less influenced by external cultures over time. This relative isolation has allowed the Baiku Yao community to preserve its traditional lifestyle and cultural practices but has also limited the introduction of new ideas and customs. Therefore, while Guangdong and Guangxi are geographically close, the Baiku Yao area does not share the rich culture of herbal soups and herbal teas seen in Guangdong. This phenomenon indirectly supports the notion of the Baiku Yao as a "cultural fossil," with its culture and traditions preserved through long-term geographical and social isolation.

This comparative analysis not only underscores the Baiku Yao’s unique utilization of edible plants but also reveals the deep-seated differences stemming from each region’s distinct geographical, ecological, and cultural contexts. By highlighting the significant roles of geographical and ecological factors in the selection and use of traditional edible plants, this study offers valuable insights into preserving and understanding the plant knowledge inherent in these cultures. Such insights are crucial for developing strategies that safeguard this invaluable cultural heritage.

## Conclusion

The Baiku Yao’s traditional knowledge and use of edible plants represent a rich tapestry of ethnobotanical wisdom, deeply intertwined with their cultural identity and survival strategies. Their diversified use of plants, including vegetables, fruits, spices, and medicinal herbs, reflects a deep understanding of local biodiversity and a sustainable approach to resource utilization. The CFSI has proven instrumental in categorizing plants based on their cultural significance, illuminating the community’s reliance on and reverence for these natural resources. However, the encroachment of modernization poses significant threats to the preservation of this knowledge. It is imperative to document, protect, and transmit these practices, not only to sustain the Baiku Yao’s cultural heritage but also to contribute to global knowledge on sustainable living and biodiversity conservation. This study serves as a vital academic reference for future research and underscores the need for concerted efforts in ethnobotanical conservation.

## Data Availability

All data generated or analyzed during this study are included in this published article.

## Abbreviations

CFSI: Cultural food significance index
QI: Quotation index
AI: Availability index
PUI: Parts used index
FUI: Frequency of utilization index
MFFI: Multifunctional food use index
TSAI: Taste score appreciation index
FMRI: Food-medicinal role index
RFC: Relative citation frequency

## References

[1] Turner NJ, Łuczaj ŁJ, Migliorini P, Pieroni A, Dreon AL, Sacchetti LE, Paoletti MG (2011). Edible and tended wild plants, traditional ecological knowledge and agroecology. Crit Rev Plant Sci.

[2] Geng YF, Yang Y, Zhang Y, Zhang LL, Wang YH (2015). Research development of food plant ethnobotany——bibliometric and mapping knowledge domains analysis based on web of science. Plant Divers Resour.

[3] Reyes-García V, Menendez-Baceta G, Aceituno-Mata L, Acosta-Naranjo R, Calvet-Mir L, Domínguez P, Garnatje T, Gómez-Baggethun E, Molina-Bustamante M, Molina M (2015). From famine foods to delicatessen: interpreting trends in the use of wild edible plants through cultural ecosystem services. Ecol Econ.

[4] Gruber K (2017). Agrobiodiversity: the living library. Nature.

[5] de Bruin S, Dengerink J, van Vliet J (2021). Urbanisation as driver of food system transformation and opportunities for rural livelihoods. Food Secur.

[6] Kookana RS, Drechsel P, Jamwal P, Vanderzalm J (2020). Urbanisation and emerging economies: issues and potential solutions for water and food security. Sci Total Environ.

[7] Laborde D, Martin W, Swinnen J, Vos R (2020). COVID-19 risks to global food security. Science.

[8] Vandebroek I, Pieroni A, Stepp JR, Hanazaki N, Ladio A, Alves RRN, Picking D, Delgoda R, Maroyi A, Van Andel T (2020). Reshaping the future of ethnobiology research after the COVID-19 pandemic. Nat Plants.

[9] Qiu R (2008). Cultural change and identity—an examination of the current status of the Bai Ku Yao culture in Nandan County. Guangxi Econ Soc Dev.

[10] Hu R, Li T, Qin Y, Liu Y, Huang Y (2022). Ethnobotanical study on plants used to dye traditional costumes by the Baiku Yao nationality of China. J Ethnobiol Ethnomed.

[11] Hu R, Hu Q, Nong Y, Luo B (2023). Ethnobotanical study on forage plants of Baiku Yao in China. Guihaia.

[12] Luo B, Hu Q, Lai K, Bhatt A, Hu R (2022). Ethnoveterinary survey conducted in Baiku Yao communities in Southwest China. Front Vet Sci.

[13] Luo B, Nong Y, Hu R (2023). *Lindera pulcherrima* var. *attenuata* leaves: a nutritious and economically promising staple food in the Baiku Yao community in China. Front Nutr.

[14] Luo B, Liu B, Zhang H, Zhang H, Li X, Ma L, Wang Y, Bai Y, Zhang X, Li J (2019). Wild edible plants collected by Hani from terraced rice paddy agroecosystem in Honghe Prefecture, Yunnan, China. J Ethnobiol Ethnomed.

[15] Pieroni A (2001). Evaluation of the cultural significance of wild food botanicals traditionally consumed in Northwestern Tuscany, Italy. J Ethnobiol.

[16] Sujarwo W, Caneva G (2016). Using quantitative indices to evaluate the cultural importance of food and nutraceutical plants: comparative data from the Island of Bali (Indonesia). J Cult Herit.

[17] Mosaddegh M, Naghibi F, Moazzeni H, Pirani A, Esmaeili S (2012). Ethnobotanical survey of herbal remedies traditionally used in Kohghiluyeh va Boyer Ahmad province of Iran. J Ethnopharmacol.

[18] Ahmad M, Sultana S, Fazl-i-Hadi S, Ben Hadda T, Rashid S, Zafar M, Khan MA, Khan MPZ, Yaseen G (2014). An ethnobotanical study of medicinal plants in high mountainous region of Chail valley (District Swat-Pakistan). J Ethnobiol Ethnomed.

[19] Tu Y, Diao Q, Zhang R, Yan G, Xiong W (2009). Analysis on feed nutritive value of hybrid *Broussonetia papyrifera* leaf. Pratacult Sci.

[20] Hu R, Xu C, Nong Y, Luo B (2023). Changes in homegardens in relocation villages, a case study in the Baiku Yao area in Southern China. J Ethnobiol Ethnomed.

[21] Wu Z, Deng X, Hu Q, Xiao X, Jiang J, Ma X, Wu M (2021). *Houttuynia cordata* thunb: an ethnopharmacological review. Front Pharmacol.

[22] Chou SC, Su CR, Ku YC, Wu TS (2009). The constituents and their bioactivities of *Houttuynia cordata*. Chem Pharm Bull.

[23] Rafiq S, Hao H, Ijaz M, Raza A (2022). Pharmacological effects of *Houttuynia cordata* Thunb (*H. cordata*): a comprehensive review. Pharmaceuticals.

[24] Yan L, Meng Q, Kim I (2011). The effects of dietary *Houttuynia cordata* and *Taraxacum officinale* extract powder on growth performance, nutrient digestibility, blood characteristics and meat quality in finishing pigs. Livest Sci.

[25] Liu S, Huang X, Bin Z, Yu B, Lu Z, Hu R, Long C (2023). Wild edible plants and their cultural significance among the Zhuang ethnic group in Fangchenggang, Guangxi, China. J Ethnobiol Ethnomed.

[26] Ding M, Shi S, Luo B (2022). Hearty recipes for health: the Hakka medicinal soup in Guangdong, China. J Ethnobiol Ethnomed.

[27] Liu Y, Liu Q, Li P, Xing D, Hu H, Li L, Hu X, Long C (2018). Plants traditionally used to make Cantonese slow-cooked soup in China. J Ethnobiol Ethnomed.

[28] Li DL, Zheng XL, Duan L, Deng SW, Ye W, Wang AH, Xing FW (2017). Ethnobotanical survey of herbal tea plants from the traditional markets in Chaoshan, China. J Ethnopharmacol.

